# Integrating metabolomics and machine learning to forecast anti-inflammatory and antioxidant activities in *D*. *officinale* leaves

**DOI:** 10.1186/s13020-025-01282-z

**Published:** 2026-01-06

**Authors:** Guoliang Zhang, Yuying Zhao, Chenlei Ru, Guangxin Luo, Zhuping Hong, Jihong Yang, Zhenhao Li

**Affiliations:** 1 Zhejiang ShouXianGu Botanical Drug Institute, Hangzhou, Zhejiang China; 2 BoYu Intelligent Health Innovation Laboratory, Hangzhou, Zhejiang China; 3 Zhejiang Key Laboratory of Biological Breeding and Exploitation of Edible and Medicinal Mushrooms, Wuyi, China

**Keywords:** *D. officinale* leaves, Metabolite dynamic profiling, Machine learning, Anti-inflammatory, Antioxidant activity

## Abstract

**Background:**

*Dendrobium officinale* (*D. officinale*) leaves, rich in bioactive compounds comparable to those in stems, remain underutilized as agricultural byproducts.

**Purpose:**

This study aims to establish an ML (machine learning)-driven metabolomic framework to evaluate seasonal variations in bioactive compounds within *D. officinale* leaves, identify germplasm-specific pharmacological activities, and determine core components driving anti-inflammatory and antioxidant effects.

**Methods:**

An integrated approach combining dynamic metabolomic profiling (UHPLC-QTOF-MS, RP-HPLC, and UPLC-QqQ-MS), in vitro bioassays (TNF-α/IL-6 suppression assays and ABTS radical scavenging assay), and ML modeling was employed.

**Results:**

Phenolics, flavonoids, terpenes, and B-vitamins peaked in October–November, while amino acids accumulated until December. Despite this, July-harvested leaves exhibited maximum anti-inflammatory and antioxidant activity. Random Forest Regression (RFR) models identified vanillic acid 4-*β*-D-glucoside, schaftoside, and rutin as key bioactive contributors, validated experimentally.

**Conclusion:**

This ML-enhanced metabolomic strategy advances the quality assessment and germplasm optimization of *D. officinale* leaves by linking dynamic phytochemical profiles to bioactivity. The identification of July as the optimal harvest period and critical bioactive compounds underscores the approach’s utility in nutraceutical and pharmaceutical applications, promoting sustainable utilization of agricultural byproducts.

**Supplementary Information:**

The online version contains supplementary material available at 10.1186/s13020-025-01282-z.

## Introduction

*Dendrobium officinale (D. officinale)*, a prominent species within the Dendrobium genus, has been utilized as a Chinese herbal medicine and functional food in China and other Asian countries for over a millennium. Historically revered as “the first of the nine immortal herbs” in Chinese traditional medicine [1]. Currently, this orchid species is extensively cultivated across southern Chinese provinces, including Zhejiang, Yunnan, Guizhou, and Fujian [2]. While the *Chinese Pharmacopoeia* exclusively authorizes the use of *D. officinale* stems for medicinal applications, the fresh leaves—constituting approximately 50% of total plant biomass—are systematically discarded as agricultural byproducts [2, 3]. Although scientific research predominantly focuses on *D. officinale* stems, which contain abundant polysaccharides, flavonoids, and bioactive metabolites, emerging evidence confirms that the leaves harbor comparable therapeutic constituents including polysaccharides, flavonoid derivatives, vitamins, and essential amino acids [4–6]. Pharmacological investigations have demonstrated that *D. officinale* leaf extracts exhibit multifunctional biological activities, including anti-inflammatory, antioxidant, antihypertensive, and antitumor effects, positioning them as valuable resources for pharmaceutical development, nutraceutical formulations, and food additive applications [7, 8]. These findings have consequently propelled research into optimizing utilization strategies for this currently undervalued plant material.

Contemporary phytochemical analysis has become instrumental in characterizing *D. officinale* leaves, owing to its operational efficiency and technical simplicity. Current analytical approaches include gas chromatography (GC) [9], high-performance liquid chromatography (HPLC) [6], ultra-performance liquid chromatography mass spectrometry (UPLC-MS) [6], and nuclear magnetic resonance (NMR) [10]. Nevertheless, existing research predominantly focuses on polysaccharides, flavonoids, and phenolic compounds, with limited comprehensive investigations into germplasm variability across developmental stages. Free amino acid quantification serves as a critical parameter for evaluating functional food quality. HPLC methods with precolumn derivatization have emerged as the gold standard for amino acid analysis due to their superior sensitivity and selectivity, having been successfully implemented in diverse matrices including beverages, apicultural products, meat, and nutraceuticals [11–14]. Vitamins play essential roles in regulating human metabolic processes and maintaining physiological homeostasis. Liquid chromatography-mass spectrometry (LC–MS) has demonstrated particular efficacy in detecting B-complex vitamins across food, pharmaceutical, and biological samples [15–17].

Recent advances in artificial intelligence (AI) and machine learning (ML) have revolutionized data analysis in complex biological systems, offering unprecedented opportunities for traditional medicine research [18–20]. AI techniques excel at processing high-dimensional datasets, identifying patterns invisible to conventional statistical methods, and establishing predictive models that correlate chemical profiles with biological activities. In the realm of Chinese herbal medicines, AI has proven invaluable for quality control, mechanistic studies, treatment optimization, and diagnostic applications. Particularly in cheminformatics-driven quality assessment, machine learning algorithms can integrate diverse analytical data to predict therapeutic efficacy based on bioactive constituent profiles, addressing the critical need for rapid, comprehensive quality evaluation systems in phytopharmaceuticals [21, 22].

Limited by the large coverage area of *D. officinale* leaves and the complex pharmacological and chemical composition, it is difficult to evaluate its quality [23, 24]. In general, the efficacy determination of *D. officinale* leaves is one of the indicators to evaluate its quality. However, this method is time-consuming, costly, and requires demanding experimental conditions [9]. Chemical analysis because of its quick and easy to operate, also widely used in quality assessment [6]. Both of them studied the quality evaluation of Chinese herbal medicines from different levels. Therefore, it may be attractive to establish a quality evaluation method based on chemical analysis to realize rapid quality evaluation of Chinese herbal medicines by integrating efficacy determination with chemical analysis. However, the chemical composition of *D. officinale* leaves is complex, including abundant small molecule compounds, nutrients and polysaccharides [25–27]. Single compound index has limitations, and multi-component comprehensive evaluation is more realistic. *D. officinale* leaves and its products are widely used to treat dermatologic disorders due to their excellent anti-inflammatory and antioxidant activities [1, 10, 28]. Therefore, small molecular compounds and nutritional components of different varieties of *D. officinale* leaves were selected as research objects in this paper, and the anti-inflammation and antioxidant activities of cells were evaluated through in vitro experiments.

**Fig. 1:**
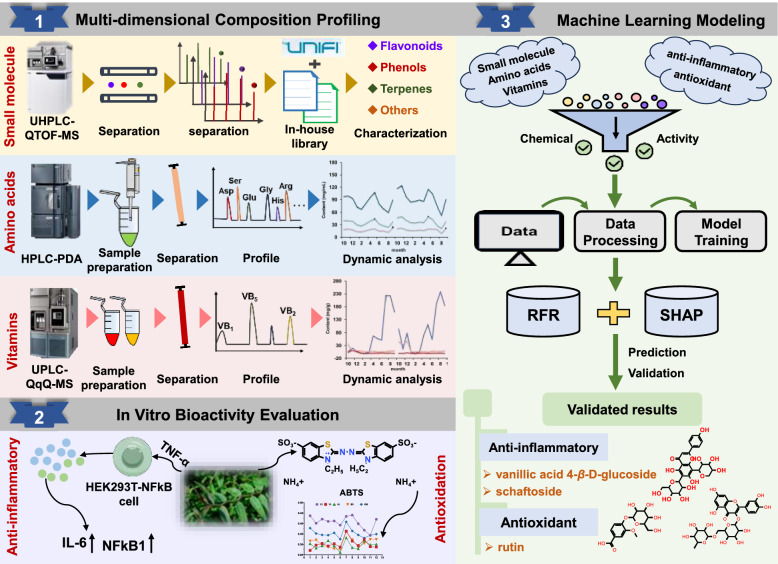
The flow chart for developing a comprehensive analytical strategy for studying metabolic profiles and pharmacological contributions of multi-component active ingredients in *D. officinale* leaves

In this study, to investigate the dynamic changes in the metabolic profiles of *D. officinale* leaves from different germplasms and elucidate the contributions of multi-component active ingredients to pharmacological effects, a comprehensive analytical strategy was implemented, the general flowchart is displayed in Fig. 1. First, a combination of advanced analytical methods, including UHPLC-QTOF-MS, RP-HPLC, and UPLC-QqQ-MS was established to achieve thorough characterization of small-molecule compounds (e.g., phenolics, flavonoids, terpenes) and nutrients (vitamins and amino acids), along with dynamic analysis of their relative contents. Subsequently, the antioxidant and anti-inflammatory activities of *D. officinale* leaves were systematically evaluated using standardized in vitro assays. Finally, a correlation model linking multi-component chemical active ingredients to pharmacodynamic quantitative parameters was developed to identify specific bioactive components. This study aims to establish a rapid and feasible analytical framework for comprehensive chemical and pharmacological profiling of *D. officinale* leaves, enabling pharmacological activity prediction based on active ingredient content. The proposed methodology holds significant potential for quality evaluation and germplasm screening of *D. officinale* leaves and analogous medicinal materials.

## Material and methods

### Materials

HPLC grade acetonitrile, formic acid and methanol (MeOH) were purchased from Merck (Darmstadt, Germany). Ultrapure water was prepared using a water purification system (Direct Q5 ultrapure water system). All other chemicals and reagents used such as ethyl alcohol, phenol, and hydrochloric acid (HCl) were analytical grade. Chloramphenicol (> 99%) was obtained from McLean Biochemical Technology Co., Ltd (Shanghai, China). The analytical standards of thiamine (VB1), riboflavin (VB2), and pantothenic acid (VB5) with high purity in the range of 98%–99%, and folic acid (> 91%) were obtained from National Institutes for Food and Drug Control. Nicotinamide mononucleotide, rutin, vanillic acid 4-*β*-D-glucoside, and schaftoside (> 95%) were purchased from Chengdu Pusi Biotechnology Co., Ltd (Chengdu, China). All amino acids standards were purchased from Waters Corporation. High glucose DMEM (Batch No. BC20230413), penicillin–streptomycin (Batch No. BC20221202), 0.25% trypsin (Batch No. BC20221202) were purchased from Bio-Channel Company (Nanjing, China). Fetal brovine serum (FBS) was from Genom (Hangzhou, China). CCK-8 detection kit and RNA extraction reagent RNAiso Plus were purchased from MCE (Shanghai, China) and ‌Takara (Beijing, China). RNA Reverse Transcription Kit and UltraSYBR Mixture were purchased from Kangwei Century Biotechnology Co., Ltd (Nanjing, China). ABTS Kit, Bright-Lumi Firefly Luciferase Reporter Assay Kit and Puromycin were all from Beyotime (Shanghai, China). TNF-a was from Sino Biological (Beijing, China), and HEK293T-NFKB1 cells were purchased from Zhejiang University (Zhejiang, China). RNAiso Plus (Takara Bio, Japan, Cat# AM91949A), reverse transcription reagent kit (Jiangsu Cowin Biotech, Cat# 28,522), UltraSYBR^®^ Mixture (Jiangsu Cowin Biotech, Cat# 07223). Superoxide dismutase kit (SOD, Cat# S0101S), malondialdehyde (MDA, Cat# S0131S), and reduced glutathione (GSH, Cat# S0053) were purchased from Beyotime Institute of Biotechnology (Shanghai, China). A total of 60 batches of *D. officinale* leaf powder, including 5 varieties, were provided by Zhejiang Shouxiangu Pharmaceutical Co., Ltd (Wuyi Zhejiang). The detailed information of the samples was available from Supplementary Table S1.

### Preparation of aqueous extract from *D. officinale* leaves

Approximately 1 g of *D. officinale* leaves powder was weighed and mixed with 30 mL of distilled water. The mixture was heated in a water bath at 100 °C for 3 h. After cooling, the solution was centrifuged at 5000 rpm for 20 min. The supernatant was collected in a dry evaporating dish and subjected to freeze-drying to obtain the aqueous extract. Subsequently, 0.2 g of the aqueous extract was dissolved in 10 mL of DMEM medium. After complete dissolution, the solution was filtered through a 0.22 µm membrane filter. The filtrate was stored at − 20 °C for further use.

### UHPLC-QTOF-MS/MS analysis

Stock solutions of chloramphenicol internal standard (10 mg/mL) was prepared by dissolving 100 mg of the chloramphenicol in 10 mL of MeOH. The accurately weighed powder of each sample was soaked in a 15 mL centrifuge tube containing 10 mL of 70% (*v/v*) MeOH and 4 µL of chloramphenicol internal standard solution. The mixture was sonicated in an ultrasound bath for 30 min (40 kHz) and then centrifuged for 10 min at 13,000 g. The supernatant was collected for UPLC-Q-TOF/MS analysis.

The UPLC-MS analysis was performed on a Waters ACQUITY UPLC system coupled with a Waters SYNAPT XS High-Definition Mass Spectrometer equipped with electrospray ionization (ESI) source (Waters Co., MA, USA). Chromatographic separations were performed on a Waters ACQUITY UPLC HSS T3 (2.1 mm × 100 mm, 1.8 µm) with the column temperature set at 25 °C. The mobile phase A was water containing 0.1% formic acid, while the mobile phase B was acetonitrile. This mobile phase system was applied with the following gradient program: 0–1 min, 5% B; 1–5 min, 5%–17% B; 5–8 min, 17%–20% B; 8–12 min, 20%–42% B; 12–13 min, 42%–55% B; 13–18 min, 55%–70% B; 18–20 min, 70%–90% B; 20–25 min, 90% B; 25–25.5 min, 90%–5% B; 25.5–28 min, 5% B. The flow rate was 0.4 mL/min, and the injection volume was 1 µL. Mass spectrometry was performed for negative mode and the parameters were set as follows: source temperature of 150 °C, desolvation gas flow of 1000 L/h at a temperature of 450 °C, capillary voltage of 2.5 kV, cone voltage of 35 V, collision energy of 20–45 eV, and scan range of *m/z* 50–1200. Data were corrected during acquisition using a reference consisting of leucine enkephalin (negative with *m/z* 554.2615).

### Determination of amino acids in RP-HPLC

0.1 g powder of each *D. officinale* leaves was weighed and mixed with 8 mL of 6 M HCl and 2 drops of phenol solution, filled with nitrogen and sealed. The mixture was incubated in an oven at 110 °C for 22 h. Cool the reaction solution to room temperature and centrifuge at 13,000 g for 10 min. Aliquots containing 100 µL of this stock solution were transferred to centrifuge tube (1.5 mL) and dried by vacuum centrifugation. Each of the dried samples were reconstituted in 100 µL of 20 mM HCl and then centrifuged for 10 min (13,000 g). Next, aliquot of 10 µL sample was placed in a derivative tube, 70 µL AccQ**‧**Fluor borate buffer was added to generate the PH environment at the range of 8.2–10.0, then an addition of 20 µL AccQ**‧**Fluor reagent was added, and the mixture was heated in the oven at 55 °C for 10 min. The supernatant was centrifuged at 13,000 g for 10 min for HPLC analysis.

Amino acids were analyzed on a Waters ACQUITY Arc HPLC system equipped with a Waters 2998 photodiode array (PDA) detector (Milford, MA, USA). A Waters AccQ**‧**Tag amino acid analysis column (3.9 mm × 150 mm, 4 µm) was used for chromatographic separation. The column temperature was 37 °C, the injection volume was 5 µL, and the flow rate was 1 mL/min. Mobile phase A consisted of eluent A (prepared from Waters AccQ**‧**Tag Eluent A concentrate), mobile phase B was acetonitrile, and mobile phase C was water. The gradient separation program was as follows: 0 min: 100% A, 0% B, 0% C; 0.5 min: 99% A, 1% B, 0% C; 18.0 min: 95% A, 5% B, 0% C; 19 min: 91% A, 9% B, 0% C; 29.5 min: 83% A, 17% B, 0% C; 33 min: 0% A, 60% B, 40% C and 36–41 min: 100% A, 0% B, 0% C. UV detection at 240 nm was used.

### Determination of water-soluble vitamins in UPLC-QqQ-MS

Preparation of standard solutions: Individual VB1, VB5, and NMN stock solutions were prepared by dissolving the standards in deionized water at concentration 0.5 mg/mL, while the folic acid stock solution needs 6 mL of 0.5% ammonia to assist dissolution. VB2 stock solution in 100 µL formic acid and water at 0.2 mg/mL concentration. All standards were stored were stored in the dark at 4 °C. The working standard solution was prepared by mixing the appropriate volume of these single standard reserve solutions in a 10 mL volumetric bottle (VB1/100 µL, VB2/VB5/500 µL, folic acid/NMN/250 µL) and diluting them to the desired concentration with deionized water.

Preparation of sample solution: 0.5 g of each sample was weighed and dissolved in an appropriate amount of water, and then fixed in a 10 mL volumetric flask. Then, aliquots containing 4 mL of this solution were transferred to 10 mL volumetric flask and diluted with ethanol to volume. After centrifugation at 13,000 g for 10 min, the supernatant was taken for analysis (20 mg/mL).

The UPLC-MS/MS system comprised of a Waters ACQUITY I-Class UPLC system (Milford, MA, USA) equipped with a Xevo TQ-S mass spectrometer, and a Waters ACQUITY UPLC HSS T3 column (2.1 mm × 100 mm, 1.8 µm). The mobile phases were water containing 0.1% formic acid (phase A) and acetonitrile containing 0.1% formic acid (phase B), and the flow rate was 0.3 mL/min. The following gradient elution program was used: 0–3 min, 1% B; 3–3.1 min, 1–5% B; 3.1–5.5 min, 5–20% B; 5.5–7.5 min, 20–70% B; 7.5–9 min, 70% B; 9–9.5 min, 70–1% B; 9.5–12 min, 1% B. The injection volume was 1 µL, and the column temperature was set to 40 °C. A multiple reaction monitoring (MRM) operated in negative mode was used for MS/MS analysis. The optimized operating parameters were as follows: collision energy, 3.5 kV; source temperature, 150 °C; desolvation temperature, 500 °C; desolvation gas flow of 1000 L/h; cone gas flow of 50 L/h. The MRM transformation and optimization parameters are provided in supplementary Table S2.

### Cell viability assay

*D. officinale* leaves aqueous extract were dissolved in Dulbecco’s modified Eagle medium (DMEM; 20 mg/mL) and passed through a 0.22-µm filter. After cell culture in 96-well plates for 6 h, 100 µL cell suspension and 100 µL phosphate-buffered saline (PBS) were added to each well. The PBS was aspirated out and added to different DMEM samples with different concentrations, and cells were cultured for another 24 h. After treatment for 24 h, the original medium was removed, and then the cells were treated with cell counting kit (CCK)-8 reagent. 90 µL of DMEM and 10 µL of CCK-8 reagent was added to each well (the wells without cells as a blank group), and the microplate pores were placed in a constant temperature incubator under the same cell culture conditions, avoiding light. After reacting for 2 h, the absorbance at a wavelength of 450 nm was measured, and cell viability was calculated according to the Eq. (1).

1 $$ \left[ {\left( {{\text{OD}}_{{\text{experimental group}}} - {\text{OD}}_{{\text{control group}}} } \right)/\left( {{\text{OD}}_{{\text{experimental group}}} - {\text{OD}}_{{\text{control group}}} } \right)} \right] \times {1}00\% $$

### ABTS radical scavenging activity assay

The antioxidant activity of the samples was tested according to the literature method with slight modifications. Firstly, *D. officinale* leaves aqueous extract were diluted with PBS into solutions with concentrations of 0.625, 1.25 and 2.5 mg/mL. The ABTS-working was stand in the dark for 16 h, and then diluted with PBS to an absorbance of 0.7 (± 0.05) at 734 nm. 10 µL of sample at different concentrations was added to 96-well plates, and 200 µL of an ABTS reaction solution was added. In addition, PBS instead of the sample was used for the blank and different concentrations of Trolox was used as the positive control. The reaction mixture was shaken and incubated for 2–6 min, and the absorbance at a 734-nm wavelength.

### Establishment of an acute gastritis mouse model and evaluation of drug efficacy

#### Grouping, modeling, and drug administration

Seventy male 6-week-old SPF-grade C57BL/6 mice were acclimatized for 1 week. Mice were randomly assigned by body weight into seven groups (n = 10/group): Control group, Model group, positive control group (39 mg/kg/day, Ranitidine), low-dose *D*. *officinale* leaves extract (200 mg/kg/day, DOL-L, extacted from the variety Y2) and high-dose (600 mg/kg/day, DOL-H, extacted from the variety Y2) groups, low-dose rutin (20 mg/kg/day, Rutin-L) and high-dose (60 mg/kg/day, Rutin-H) groups. All treatment groups received daily intragastric (i.g.) administrations for seven consecutive days; Control and Model groups received i.g. saline (equivalent volume). On day 8, acute gastritis was induced in all groups except the Control group via i.g. administration of 400 μL HCl/EtOH solution (150 mM HCl containing 60% ethanol). Animals were euthanized one hour post-induction. Gastric tissues were harvested, snap-frozen in liquid nitrogen for storage at -80 °C, or fixed in 4% paraformaldehyde (PFA).

#### Assessment of MDA and GSH levels, and SOD activity in gastric tissues

Gastric tissues were collected and homogenized in PBS at a 1:9 (*w*/*v*) tissue-to-solution ratio. Homogenates were processed according to commercial assay kit instructions to quantify MDA, GSH levels, and SOD activity.

#### Quantitative real-time PCR (qRT-PCR) analysis

Total RNA was extracted from gastric tissues of each experimental mouse group. RNA concentration and purity were determined using a NanoDrop One UV–Vis Microvolume Spectrophotometer (Thermo Fisher Scientific, USA). Complementary DNA (cDNA) was synthesized from total RNA by reverse transcription, strictly following the manufacturer’s protocol for the designated reverse transcription kit. Quantitative real-time PCR (qPCR) was performed using SYBR Green I chemistry on a CFX Connect™ Real-Time PCR Detection System (Bio-Rad, USA) to amplify target cDNAs. The relative mRNA expression levels of TNF-α, IL-6, and IL-1β, normalized to the endogenous control GAPDH, were calculated using the comparative threshold cycle method (2^−△△Ct^). Primer information shown in the Table S3.

#### Statistical analysis

Data are expressed as mean ± standard error of the mean (SEM). Statistical analyses were performed using GraphPad Prism (v.10.6.1; GraphPad Software). Intergroup differences were assessed by two-tailed Student's *t*-test for two-group comparisons or one-way analysis of variance (ANOVA). Statistical significance was defined as P < 0.05.

### Calibration model development

The chemical composition analysis results were structured into a data matrix with columns representing bioactive component concentrations (78 variables) and rows corresponding to 60 analyzed samples. To optimize model performance and training efficiency, variables containing > 50% missing values were excluded. Missing values in the remaining 61 variables were imputed with zeros, followed by mean-centered standard deviation normalization to standardize feature scales. Quantitative models linking phytochemical profiles to pharmacological indices were developed using three algorithms: (1) a linear partial least squares regression (PLSR), and (2) two nonlinear methods—least squares support vector machine (LS-SVM) and random forest regression (RFR).

PLSR constructs linear regression models by projecting predictor (X) and response (Y) matrices into a latent space, leveraging covariance structures to address multicollinearity. This method is particularly suited for datasets with correlated variables or when variables out number observations. The optimal number of principal components (${\text{N}}_{\text{pc}}$) was determined via grid search to minimize the root-mean-quared error of cross-validation (RMSECV) [29].

LS-SVM, an advanced variant of support vector machines, employs nonlinear mapping to transform input features into a high-dimensional space for improved regression performance. Optimal regularization and radial basis function (RBF) kernel parameter $\sigma^{2}$ were determined using a three-structured Parzen estimator (TPE) to maximize predictive accuracy [30, 31].

RFR [32] leverages an ensemble of decision trees trained on bootstrapped samples and random feature subsets to mitigate overfitting. Predictions are generated by averaging outputs across trees. Hyperparameter tuning focused on optimizing the number of iterations (tree count) through grid search, balancing computational efficiency and model performance.

#### Model implementation and evaluation

The dataset was partitioned into calibration and prediction sets using the sample set partitioning based on joint X–Y distances (SPXY) method to ensure representative distributions of chemical and bioactivity variance. Model robustness was assessed via threefold cross-validation on the calibration set, with performance metrics (mean ± SD of three iterations) reported. Model performance was evaluated using root mean squared error (RMSE) and coefficients of determination (${\text{R}}^{2}$) for calibration (${{\text{R}}_{\text{c}}}^{2}$), cross-validation (${{\text{R}}_{\text{cv}}}^{2}$), and prediction (${{\text{R}}_{\text{p}}}^{2}$), with lower RMSE and higher ${\text{R}}^{2}$ indicating superior performance.

To assess model stability, the bootstrap method (500 iterations) was applied, randomly resampling 90% of the calibration set for model retraining. Prediction stability was quantified via relative standard deviation (RSD) of 1,500 predictions (500 iterations × 3 cross-validation folds) for each test sample.

All analyses were implemented in Python (v3.8.5) using the Numpy (v1.23.5) and Scikit-learn (v1.1.3) libraries. Hyper-parameter optimization for LS-SVM utilized the hyperopt (v0.2.5) library.

## Results

### Metabolomic profiles of *D. officinale* leaves

**Fig. 2:**
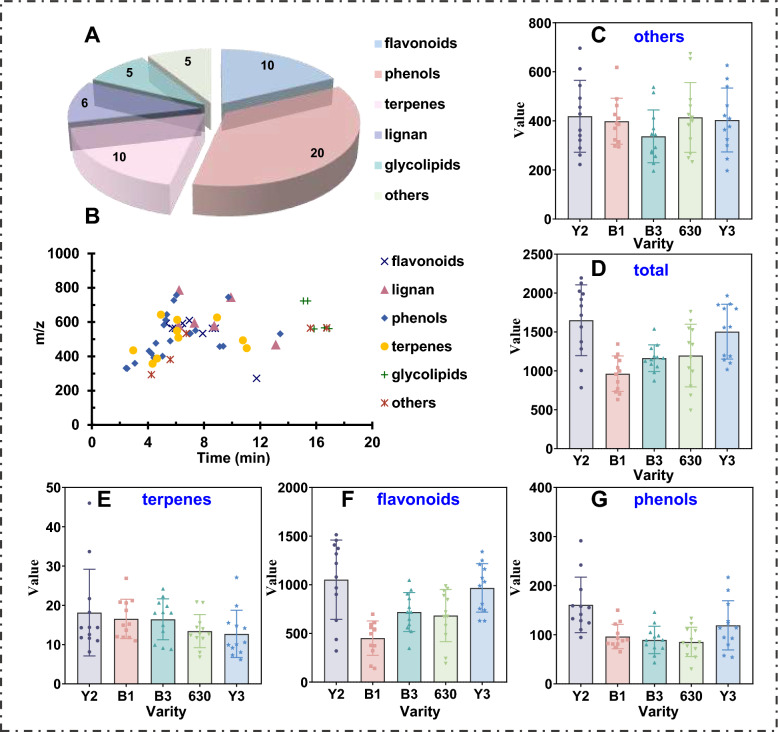
Statistics and comparison of characteristic components from different varieties of *D. officinale* leaves. Venn diagram showing the different types of compounds identified (**A**); scatter plots (*t*_R_ VS *m/z*) of the compounds characterized from *D. officinale* leaves, by showing the different classes (**B**); Summary of relative content of different types of compounds (**C**–**G**)

The versatile data processing platform UNIFI 1.9, by searching the incorporated in-house *D. officinale* leaves library and matching the high-accuracy negative-mode CID-MS^E^ data, achieved a comparative analysis for structural characterization of *D. officinale* leaves. Based on the established workflows, a total of 56 compounds were tentatively identified (Table S4), and the number of phenols, flavonoids, and terpenes compounds were the largest among them, accounting for 20, 10, and 10%, respectively (Fig. 2A). To provide an intuitive characterization of the chemical composition of *D. officinale* leaves, scatter plots of retention time and mass number of different compounds were prepared (Fig. 2B). In terms of the total amount of various types of compounds, Y2 variety contains more abundant compounds, followed by Y3 variety (Fig. 2C–G).

The relative contents of each component in *D. officinale* leaves were calculated according to Eq. (2). All the relative contents are shown in Supplementary Table S5.

2 $$ \frac{{C_{r} \times A_{s} \times V}}{{A_{r} \times W}} $$

In the equation, C_r_ is the internal standard concentration; A_s_ is the response value of the sample; V is the volume of 70% methanol; A_r_ is the internal standard response; W is the weight of sample.

### Analysis of dynamic changes of metabolites in *D. officinale* leaves at different harvesting periods

**Fig. 3:**
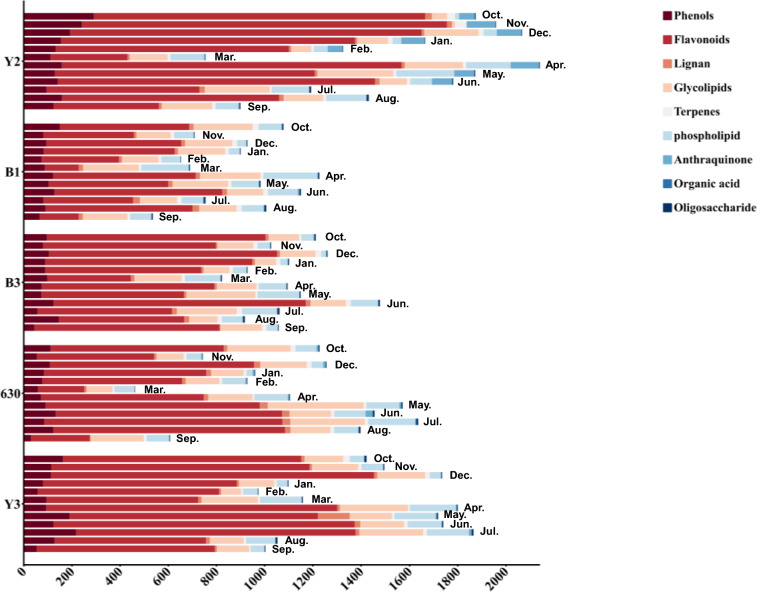
Histogram of the dynamic changes of metabolites of *D. officinale* leaves in different months

To illustrate the proportion and dynamic changes of various secondary metabolites across harvest periods, the average content was used to draw a relative content histogram (Fig. 3). Significant seasonal differences were observed in the relative contents of most metabolites, and the detailed dynamic changes were further elucidated.

Notably, the relative content of total phenolics was marginally higher in variety Y2, with no significant differences observed in other varieties. Phenolic acids peaked in November and June–August, correlating with UV exposure and temperature shifts. Flavonoids, reached maximal levels in November but declined in May. Terpenoids, dominated by iridoids, surged in October, potentially linked to cold stress responses. Glycolipids and phospholipids showed less seasonal variation, suggesting stable biosynthesis. The relative content of each type of compound is shown in the Table S5.

### Analysis of amino acid and vitamin

**Fig. 4:**
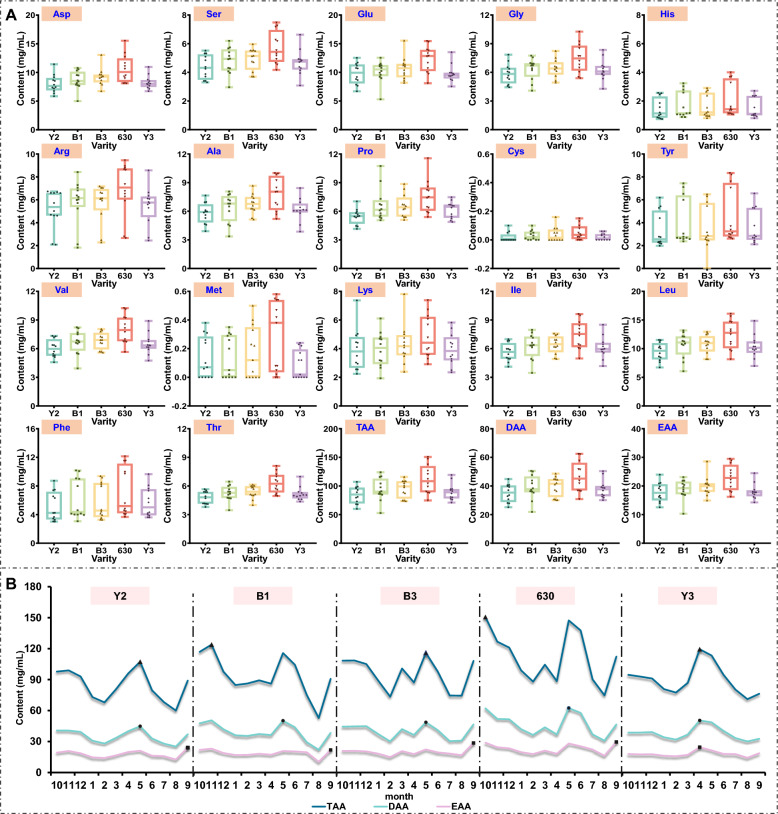
The total content box diagram of different types of amino acids in different varieties (**A**) and the dynamic change line chart of amino acids in different months (**B**)

An RP-HPLC method was validated for quantifying 17 amino acids in *D. officinale* leaves, demonstrating good linearity (*R*^2^ > 0.993), precision (RSD < 4.91%), and recovery (Table S6). Analysis of five varieties revealed distinct amino acid profiles (Fig. S1). Glutamine derivatives dominated, with leucine (10.73 mg/g), glutamate (10.55 mg/g), and aspartic acid (8.96 mg/g) as the most abundant, whereas sulfur-containing amino acids (cysteine: 0.03 mg/g; methionine: 0.08 mg/g) were minimal or absent in certain varieties. Notably, variety 630 exhibited the highest total amino acid content, delicious amino acids, and essential amino acids in December, differing significantly from other varieties (Fig. 4A, Table S7). Seasonally, total amino acids increased from August to December, correlating with declining temperatures (Fig. 4B).

**Fig. 5:**
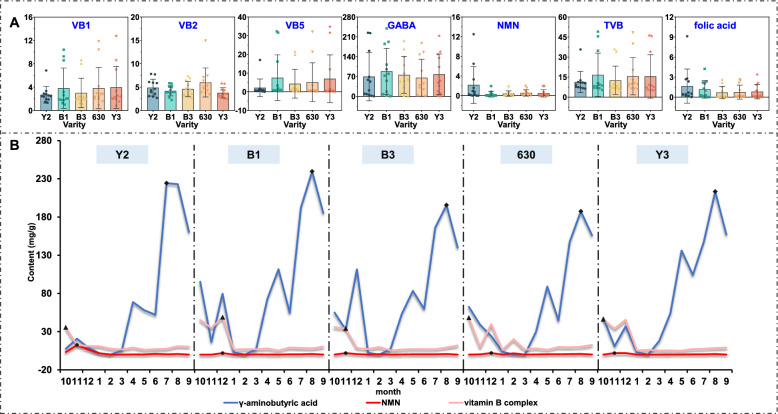
The total content box diagram of vitamin B complex in different varieties (**A**) and the dynamic change line chart in different months (**B**)

RP-HPLC analysis of five vitamins (B1, B2, B5, NMN, folic acid) highlighted inter-varietal and temporal disparities (Fig. 5, Table S8). Variety B1 displayed the highest total B-vitamins, driven by variety 630 and Y3. Variety Y2 lacked VB5, whereas uniquely accumulated folic acid and NMN (Fig. 5A). The total amount of B vitamins peaked in October–December, while folic acid declined significantly after August, and NMN showed temporal stability (Fig. 5B).

### Dynamic change analysis of anti-inflammatory activity

**Fig. 6:**
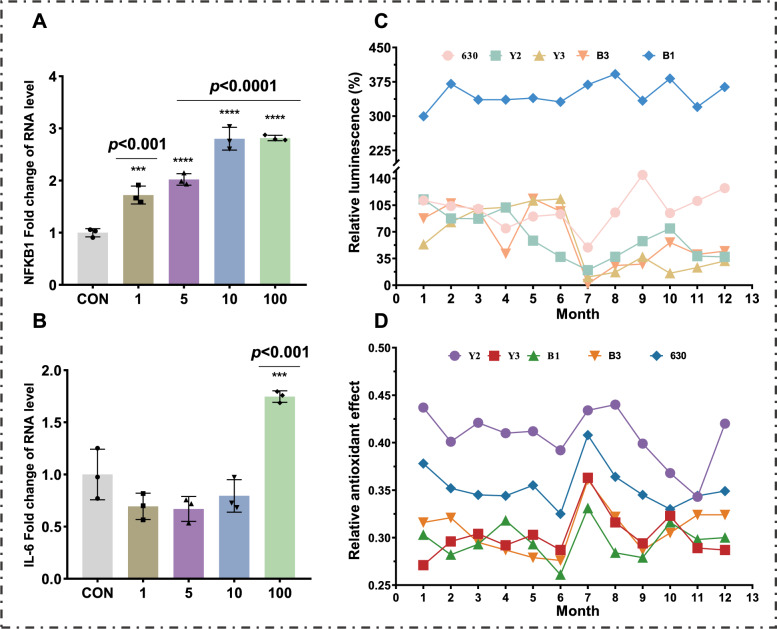
Effects of different concentrations of TNF-a on the expression of inflammation-related factors in HEK293T-NFKB1 cells (**A**, **B**); Anti-inflammatory activity of HEK293T-NFKB1 cells induced by TNF-a (**C**); Antioxidant activity detection (**D**)

To establish an inflammatory model, TNF-α-induced HEK293T-NFKB1 cells were utilized. The cell line was donated for use by the Pharmaceutical College of Zhejiang University. RT-qPCR analysis confirmed that TNF-α (100 ng/mL) significantly upregulated IL-6 (P < 0.001) and NFκB1 (P < 0.0001) mRNA levels (Fig. 6A-6B). Subsequent testing of 60 samples (crude drug concentration: 2.5 mg/mL) revealed distinct varietal differences in anti-inflammatory activity. Samples from varieties 630, Y3, and B3 reduced fluorescein readings by > 30% compared to the model group (Fig. 6C). Repeat validation (Fig. S2) confirmed activity retention in variety B3, whereas Y3 and B3 displayed inconsistencies, suggesting batch-specific bioactivity. Notably, all five varieties showed maximal anti-inflammatory effects in July (Fig. 6C).

### Dynamic change analysis of antioxidant activity

Prior to antioxidant evaluation, cytotoxicity of 60 batches was assessed via CCK-8 assay (Fig. S3-S7). HEK293T-NFKB1 cell viability remained above 90% at crude drug concentrations ≤ 1.25 mg/mL, validating assay safety across the tested range. Due to substantial variability in extraction efficiency, all samples were standardized to a crude drug concentration of 2.5 mg/mL to ensure comparability.

ABTS radical scavenging assays revealed pronounced seasonal and varietal differences in antioxidant activity (Fig. 6D). Variety Y2 demonstrated the highest annual antioxidant capacity, surpassing other varieties. All varieties exhibited peak activity in July, with activity declining progressively through August to September.

### ML-driven component-efficacy interpretation

#### Model performance and key predictors

**Fig. 7:**
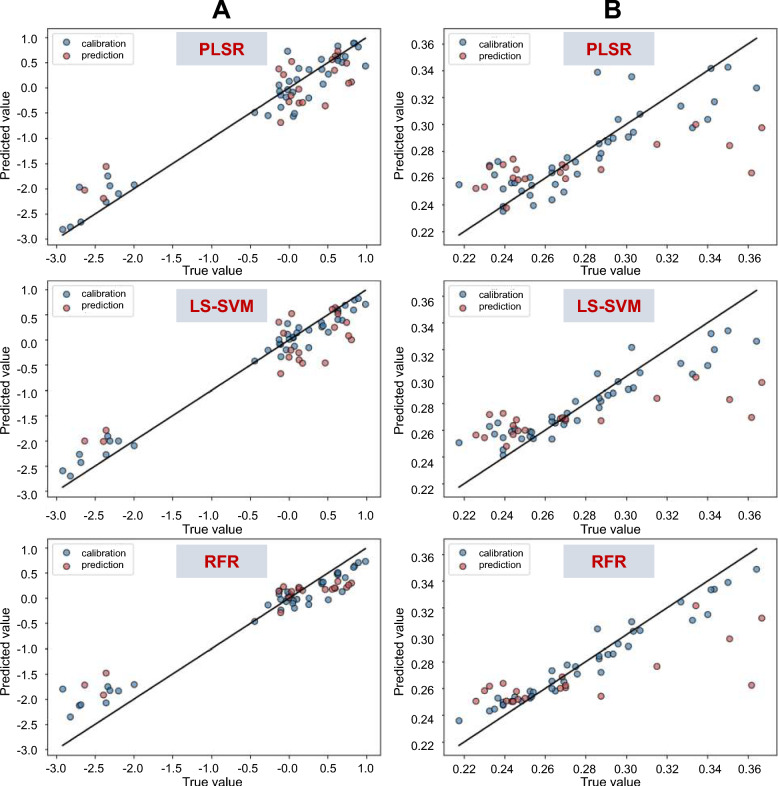
Predicted versus measured pharmacological indexes on prediction sets using the calibration models. Anti-inflammatory models (**A**); Antioxidant models (**B**)

**Fig. 8:**
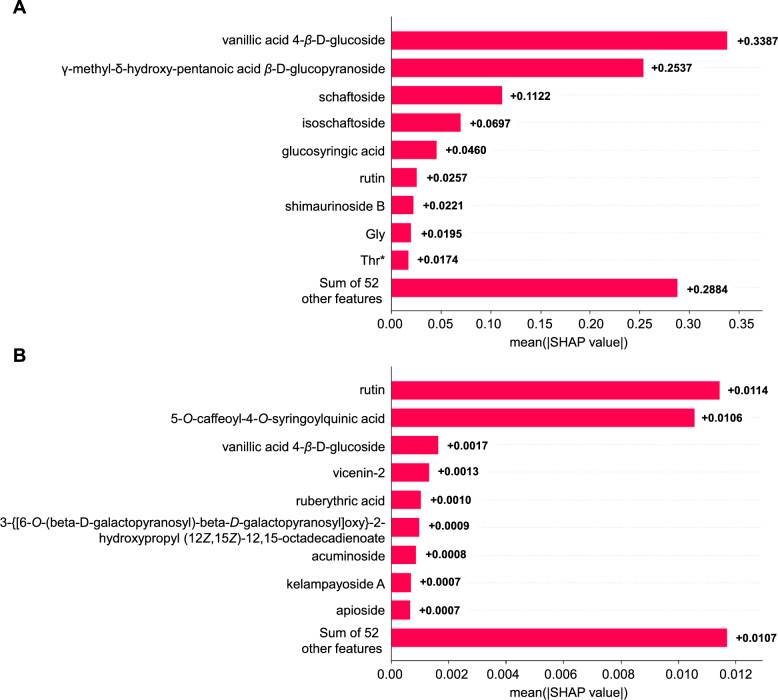
Feature importance bar plot description of SHAP values derived from RFR models. Anti-inflammatory index (**A**); Antioxidant index (**B**)

The statistical parameters of the pharmacological indices derived from the SPXY algorithm are presented in Table 1. For the anti-inflammatory index, the RFR model exhibited superior performance (${{\text{R}}_{\text{p}}}^{2}$=0.809) compared to PLSR (${{\text{R}}_{\text{p}}}^{2}$=0.671) and LS-SVM (${{\text{R}}_{\text{p}}}^{2}$=0.718), with predicted vs. measured values showing strong linearity (Table 2; Fig. 7A). SHAP analysis identified A-2 (vanillic acid 4-*β*-D-glucoside), H, B-3 (schaftoside), and B-4 as dominant contributors to anti-inflammatory effects (SHAP > 0.05, Fig. 8A). In contrast, models for antioxidant activity performed poorly: RFR achieved ${{\text{R}}_{\text{p}}}^{2}$=0.421 (Table 2), with B-6 (rutin) marginally influencing outcomes (SHAP > 0.01, Fig. 8B).

**Table 1:** The statistics pf the pharmacological indices in the calibration and prediction sets

Data set	Sample size	Indices	Mean	Max	Min	Standard deviation
Calibration set	40	Antioxidant	0.279	0.364	0.218	0.037
		Anti-inflammatory	-0.336	0.984	-2.918	1.211
Prediction set	20	Antioxidant	0.275	0.367	0.226	0.045
		Anti-inflammatory	-0.105	0.803	-2.636	1.038

**Table 2:** Model performance of the antioxidant index and anti-inflammatory index

Indices	Model	Hyper-parameter	${{R}_{c}}^{2}$	RMSEC	${{R}_{cv}}^{2}$	RMSECV	${{R}_{p}}^{2}$	RMSEP
Antioxidant	PLS	1.333	0.693	0.020	0.515	0.025	0.303	0.037
	LS-SVM	(90.314, 80.588)	0.805	0.016	0.577	0.024	0.338	0.036
	RFR	140	0.923	0.010	0.376	0.029	**0.421**	0.034
Anti-inflammatory	PLS	10	0.889	0.294	0.627	0.717	0.671	0.590
	LS-SVM	(97.012, 39.888)	0.967	0.220	0.702	0.657	0.718	0.550
	RFR	110	0.914	0.351	0.658	0.703	**0.809**	0.447

#### Validation of key anti-inflammatory and anti-oxidative components in vitro

**Fig. 9:**
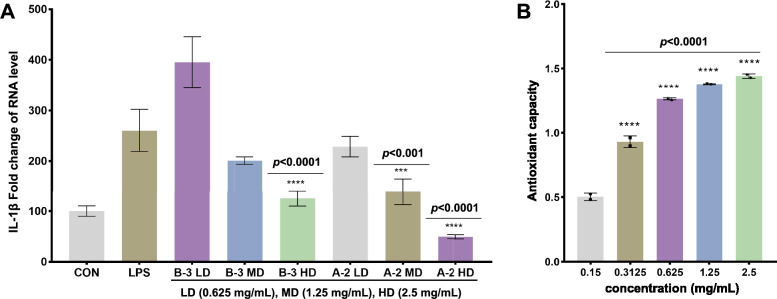
The inhibition of IL-6 expression by different doses of schaftoside (B-3 in Fig. 8A) and vanillic acid-4-*β*-D-glucoside (A-2 in Fig. 8A) (**A**). Determination of antioxidant activity of rutin (B-6 in Fig. 8B) (**B**)

Chemometric comparison of reference standards confirmed A-2, B-3, and B-6 as vanillic acid 4-*β*-D-glucoside, schaftoside, and rutin, respectively. In vitro validation using TNF-α-induced HEK293T-NFKB1 cells demonstrated dose-dependent anti-inflammatory effects: schaftoside (low/high doses) and vanillic acid 4-*β*-D-glucoside (medium/high doses) significantly suppressed inflammatory markers (P < 0.001 vs. model group; Fig. 9A). Rutin exhibited concentration-dependent ABTS radical scavenging (0.15–2.5 mg/mL; Fig. 9B), aligning with computational predictions.

#### Validating the protective effects of *D. officinale* leaves extract and rutin on oxidative stress and inflammation in murine acute gastritis

**Fig. 10:**
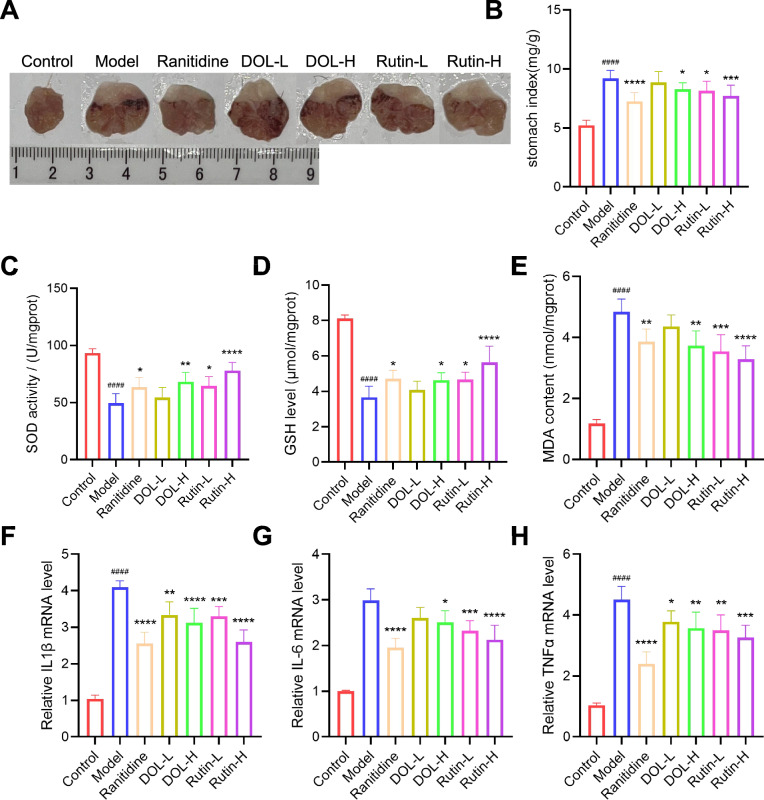
Effects of *D. officinale* leaves extract and rutin on oxidative stress and inflammation. **A** Representative images of the stomach inner layer. **B** Stomach index (n = 10). **C**–**E** Measurement of SOD, GSH, and MDA levels in gastric mucosa (n = 5). (**F**–**H**) The relative mRNA expression levels of proinflammatory cytokines (IL-1β, IL-6, and TNF-α, n = 5) in gastric mucosa. ^####^P < 0.0001 vs. Control; ^*^P < 0.05, ^**^P < 0.01, ^***^P < 0.001, ^****^P < 0.0001 vs. Model

To further verify the prediction, in vivo validation studies were undertaken by establishing an acute gastritis model. Controls exhibited intact pale-pink mucosa with defined rugae, while the model group displayed severe hemorrhagic, edematous mucosa (Fig. 10A-B). Pretreatment with *D. officinale* leaves extract (DOL-H, 600 mg/kg/day) or rutin (Rutin-L, 20 mg/kg/day; Rutin-H, 60 mg/kg/day) significantly ameliorated mucosal damage, reducing hemorrhage, congestion, and stomach index (P < 0.05 vs. Model); reversed impaired oxidative status by restoring depleted SOD activity and GSH levels while lowering elevated MDA (P < 0.05 vs. Model, Fig. 10C-E); and suppressed gastric pro-inflammatory cytokine elevations (IL-1β, IL-6, TNF-α, P < 0.05 vs. Model, Fig. 10F-H).

## Discussion

This study presented a comprehensive analysis of the dynamic metabolic profiles and pharmacological activities of *D. officinale* leaves across five varieties and harvesting periods. Integrating ML-based predictive modeling with experimental validation, we identified critical bioactive compounds driving anti-inflammatory and antioxidant effects, offering novel insights into the seasonal optimization of this underutilized medicinal resource.

The temporal metabolomic profiling revealed significant fluctuations in 56 secondary metabolites, aligning with prior studies on environmental stress-induced phytochemical accumulation in medicinal plants [2, 6]. Phenolic acids and flavonoids, key contributors to antioxidant capacity, dominant in Y2 and Y3 varieties, peaked in October–November (Fig. 3). However, maximal metabolite content did not translate to optimal bioactivity, July harvests surprisingly showed peak anti-inflammatory and antioxidant capacities. We attribute this paradox to three synergistic mechanisms: 1) Seasonal metabolic activation: Increased ultraviolet (UV) radiation and temperature fluctuations promoted biosynthesis of bioactive compounds [33, 34]. In addition, summer-elevated *β*-glucosidase activity catalyzes the hydrolysis of rutin (a flavonoid *O*-glycoside) into its quercetin aglycone, potentiating Nrf2 pathway activation-a master regulator of antioxidant response genes that mitigates oxidative stress [35]. Significantly, both quercetin and its structural analog schaftoside (a C-glycosyl flavonoid) suppress inflammation through direct inhibition of TLR4/MyD88 complex formation [36]. 2) Secondary metabolic allocation dynamics: The reduced metabolite concentrations observed in July suggest preferential channeling of photoassimilates toward vegetative growth and reproductive development. Conversely, autumnal accumulation of secondary metabolites [37]. 3) Dynamic metabolite interactions: July's unique phytochemical profile enhances synergistic efficacy: Heat-stable vitamins stabilize phenoxyl radicals during ABTS scavenging, whereas autumnal vitamins decline diminishes this effect. Furthermore, lower glutamate levels in July reduce competitive inhibition of phenolic uptake via LAT-1 transporters [38, 39]. These findings establish functional bioactivity indices as superior harvest timing determinants versus maximal accumulation metrics, echoing reports in Ginkgo biloba where seasonal abiotic stressors enhance bioefficacy through metabolic reprogramming rather than primary metabolite amplification [40]. The amino acid profiles (Fig. 4A), dominated by glutamate and aspartate, suggest *D. officinale* leaves’ potential as functional food ingredients, given their roles in neurotransmission and immune regulation [41, 42]. The September surge in essential amino acids may reflect a combined strategy of resource allocation during reproductive growth and early cold acclimation, where plants accumulate nitrogenous compounds both to support phenological transitions (e.g., flowering) and to mitigate oxidative stress triggered by seasonal environmental fluctuations (e.g., temperature drops or drought) [43]. Furthermore, heat-sensitive vitamins like B2 and B5, which declined post-August (Fig. 5B), may synergize with phenolics to amplify antioxidant effects [44, 45].

The integration of ML algorithms represents a methodological leap over traditional linear models [46, 47], enabling accurate prediction of bioactivity from complex phytochemical profiles. Our ML-driven approach highlights the superiority of the RFR model in predicting anti-inflammatory activity (${{\text{R}}_{\text{p}}}^{2}$=0.809), outperforming PLSR and LS-SVM. The identification of vanillic acid 4-*β*-D-glucoside and schaftoside as key contributors aligns with their established roles in inflammation (Fig. 8A-B). Vanillic acid derivatives are established NF-κB inhibitors [48], corroborating their role in suppressing TNF-α-induced IL-6 expression. Schaftoside, a flavonoid glycoside, demonstrates dose-dependent anti-inflammatory effects consistent with its inhibition of cytokine cascades in keratinocytes [49]. Rutin, despite its lower SHAP value in antioxidant predictions, demonstrated concentration-dependent ABTS scavenging (Fig. 9B), likely due to its phenolic hydroxyl groups stabilizing radical intermediates. Based on anti-inflammatory and antioxidant assays in vitro, we primarily validated the model-predicted characteristic bioactive ingredients, confirming the predictive capability of our framework. Critically, both the *D. officinale* leaves (600 mg/kg) and rutin (20/60 mg/kg) significantly ameliorated gastric mucosal damage (Figs. 10A-B), restored gastric tissue antioxidant defenses (elevated SOD activity and GSH levels; reduced MDA; Figs. 10C-E), and effectively suppressed pro-inflammatory cytokine expression (TNF-α, IL-1β, and IL-6; Figs. 10F-H). This multi-tiered evidence validates the accuracy and physiological relevance of our ML-guided framework.

Furthermore, while our study successfully pinpointed vanillic acid 4-*β*-D-glucoside, schaftoside, and rutin as pivotal bioactive compounds using SHAP analysis, the relatively narrow set of highlighted active ingredients may reflect the model's prioritization of dominant individual predictors. To address this limitation, future iterations of the ML framework will incorporate metabolite interaction networks and joint SHAP dependency analysis, which could reveal synergistic contributions from minor components not captured by the current feature importance ranking.

Critically, our results challenge the conventional emphasis on metabolite quantity alone for quality evaluation. Instead, we advocate for a holistic approach that integrates seasonal dynamics, compound interactions, and bioactivity. The exceptional bioactivity of July-harvested leaves, coupled with the identification of key bioactive contributors, underscores the necessity of transitioning from isolated concentration thresholds to synergy-driven quality evaluation metrics. This paradigm shift from static quantification to dynamic multi-component profiling, validated by ML-driven chemometrics and pharmacological assays, redefines optimal harvesting protocols and germplasm screening criteria for herbal matrices, prioritizing bioactivity coherence over conventional compositional maximalism.

## Conclusion

In conclusion, this ML-enhanced framework bridges traditional phytochemical analysis with predictive analytics, offering a transformative approach for quality evaluation of *D. officinale* leaves and analogous medicinal plants. By integrating dynamic metabolomics, pharmacological assays, and RFR modeling, we identified key bioactive components and seasonal/germplasm optimization windows. The methodology bridges traditional phytochemical analysis with AI-driven predictive analytics, offering a scalable framework for analogous medicinal plants.

## Supplementary Information

Supplementary material 1. Table S1 Samples information of *D. officinale* leaves. Table S2 Precursor/Product ion pairs and parameters for MRM of compounds used in this study. Table S3 Sequence of qPCR primers for RNA detection. Table S4 Identification list of secondary metabolites of *D. officinale*. Table S5 Relative content of secondary metabolites in leaves of *D. officinale* (n=2). Table S6 Linear regression data, precision, and repeatability for 17 amino acids. Table S7 The content of 17 kinds of amino acids in 60 batches of *D. officinale* leaves (mg·g^-1^, n=2). Table S8 Vitamin content of 60 batches of *D. officinale* leaves (μg·g^-1^, n=2). Figure S1 Amino acid determination chromatogram (1: Asp; 2: Ser; 3: Glu; 4: Gly; 5: His; 6: Arg; 7: Thr; 8: Ala; 9: Pro; 10: Cys; 11: Tyr; 12: Val; 13: Met; 14: Lys; 15: Ile; 16: Leu; 17: Phe). Figure S2 In vitro validation results of anti-inflammatory activity. Figure S3 Effect of variety 630 on the activity of HEK-293T-NFkB cells. Figure S4 Effect of variety Y2 on the activity of HEK-293T-NFkB cells. Figure S5 Effect of variety Y3 on the activity of HEK-293T-NFkB cells. Figure S6 Effect of variety B1 on the activity of HEK-293T-NFkB cells. Figure S7 Effect of variety B3 on the activity of HEK-293T-NFkB cells

## Data Availability

The original contributions presented in the study are included in the article/supplementary material, further inquiries can be directed to the corresponding authors. Metabolic data of the present study were deposited in the MetaboLights repository (Accession: MTBLS12787 and MTBLS12784).

## Abbreviations

GC: Gas chromatography
HPLC: High-performance liquid chromatography
UPLC-MS: Ultra-performance liquid chromatography mass spectrometry
NMR: Nuclear magnetic resonance
LC–MS: Liquid chromatography-mass spectrometry
AI: Artificial intelligence
ML: Machine learning
DMEM: Dulbecco’s modified Eagle medium
PBS: Phosphate-buffered saline
CCK: Cell counting kit
PLSR: Partial least squares regression
LS-SVM: Least squares support vector machine
RFR: Random forest regression
RMSE: Root mean square error
GSH: Reduced glutathione
SOD: Superoxide dismutase
MDA: Malondialdehyde
