# Supplementary material for: Integrating metabolomics and machine learning to forecast anti-inflammatory and antioxidant activities in D. officinale leaves
Source: Chin Med. 2026 Jan 6;21:8. doi: 10.1186/s13020-025-01282-z (PMC12771946; doi:10.1186/s13020-025-01282-z)
Supplement: Supplementary file 1 — Supplementary material 1. Table S1 Samples information of D. officinale leaves. Table S2 Precursor/Product ion pairs and parameters for MRM of compounds used in this study. Table S3 Sequence of qPCR primers for RNA detection. Table S4 Identification list of secondary metabolites of D. officinale. Table S5 Relative content of secondary metabolites in leaves of D. officinale (n=2). Table S6 Linear regression data, precision, and repeatability for 17 amino acids. Table S7 The content of 17 kinds of amino acids in 60 batches of D. officinale leaves (mg·g-1, n=2). Table S8 Vitamin content of 60 batches of D. officinale leaves (μg·g-1, n=2). Figure S1 Amino acid determination chromatogram (1: Asp; 2: Ser; 3: Glu; 4: Gly; 5: His; 6: Arg; 7: Thr; 8: Ala; 9: Pro; 10: Cys; 11: Tyr; 12: Val; 13: Met; 14: Lys; 15: Ile; 16: Leu; 17: Phe). Figure S2 In vitro validation results of anti-inflammatory activity. Figure S3 Effect of variety 630 on the activity of HEK-293T-NFkB cells. Figure S4 Effect of variety Y2 on the activity of HEK-293T-NFkB cells. Figure S5 Effect of variety Y3 on the activity of HEK-293T-NFkB cells. Figure S6 Effect of variety B1 on the activity of HEK-293T-NFkB cells. Figure S7 Effect of variety B3 on the activity of HEK-293T-NFkB cells [file 13020_2025_1282_MOESM1_ESM.docx]

***<Chinese Medicine>***

Additional file 1

**Integrating Metabolomics and Machine Learning to Forecast Anti-Inflammatory and Antioxidant Activities in D. officinale Leaves**

Guoliang Zhang^1,#^, Yuying Zhao^1,#^, Chenlei Ru^1^, Guangxin Luo^1^, Zhuping Hong^1^, Jihong Yang^1,2,3,^*, Zhenhao Li^1,2,3,^*

^#^ These authors contributed equally to this work.

1 Zhejiang ShouXianGu Botanical Drug Institute, Hangzhou, Zhejiang, China

2 BoYu Intelligent Health Innovation Laboratory, Hangzhou, Zhejiang, China

3 Zhejiang Key Laboratory of Biological Breeding and Exploitation of Edible and Medicinal Mushrooms, Wuyi, China

* Correspondence:

Zhenhao Li:

Affiliation: Zhejiang ShouXianGu Botanical Drug Institute, Hangzhou

E-mail: zhenhao6@126.com

Jihong Yang:

Affiliation: Zhejiang Key Laboratory of Biological Breeding and Exploitation of Edible and Medicinal Mushrooms, Jinhua

E-mail: 11219021@zju.edu.cn

**Contents**

**Table S1** Samples information of *D. officinale* leaves.

**Table S2** Precursor/Product ion pairs and parameters for MRM of compounds used in this study.

**Table S3** Sequence of qPCR primers for RNA detection.

**Table S4** Identification list of secondary metabolites of *D. officinale*.

**Table S5** Relative content of secondary metabolites in leaves of *D. officinale* (n = 2).

**Table S6** Linear regression data, precision, and repeatability for 17 amino acids.

**Table S7** The content of 17 kinds of amino acids in 60 batches of *D. officinale* leaves (mg·g^-1^, n = 2).

**Table S8** Vitamin content of 60 batches of *D. officinale* leaves (μg·g^-1^, n = 2).

**Figure S1** Amino acid determination chromatogram (1: Asp; 2: Ser; 3: Glu; 4: Gly; 5: His; 6: Arg; 7: Thr; 8: Ala; 9: Pro; 10: Cys; 11: Tyr; 12: Val; 13: Met; 14: Lys; 15: Ile; 16: Leu; 17: Phe).

**Figure S2** In vitro validation results of anti-inflammatory activity.

**Figure S3** Effect of variety 630 on the activity of HEK-293T-NFkB cells.

**Figure S4** Effect of variety Y2 on the activity of HEK-293T-NFkB cells.

**Figure S5** Effect of variety Y3 on the activity of HEK-293T-NFkB cells.

**Figure S6** Effect of variety B1 on the activity of HEK-293T-NFkB cells.

**Figure S7** Effect of variety B3 on the activity of HEK-293T-NFkB cells.

Table S1 Samples information of *D. officinale* leaves

| NO. | Sample number | Month | Varity |
| --- | --- | --- | --- |
| 1 | Y2Y-10 | 10 | Y2 D123 (Y2) |
| 2 | Y2Y-11 | 11 |  |
| 3 | Y2Y-12 | 12 |  |
| 4 | Y2Y-1 | 1 |  |
| 5 | Y2Y-2 | 2 |  |
| 6 | Y2Y-3 | 3 |  |
| 7 | Y2Y-4 | 4 |  |
| 8 | Y2Y-5 | 5 |  |
| 9 | Y2Y-6 | 6 |  |
| 10 | Y2Y-7 | 7 |  |
| 11 | Y2Y-8 | 8 |  |
| 12 | Y2Y-9 | 9 |  |
| 13 | B1Y-10 | 10 | Black 1 A037 (B1) |
| 14 | B1Y-11 | 11 |  |
| 15 | B1Y-12 | 12 |  |
| 16 | B1Y-1 | 1 |  |
| 17 | B1Y-2 | 2 |  |
| 18 | B1Y-3 | 3 |  |
| 19 | B1Y-4 | 4 |  |
| 20 | B1Y-5 | 5 |  |
| 21 | B1Y-6 | 6 |  |
| 22 | B1Y-7 | 7 |  |
| 23 | B1Y-8 | 8 |  |
| 24 | B1Y-9 | 9 |  |
| 25 | B3Y-10 | 10 | Black 3 A039 (B3) |
| 26 | B3Y-11 | 11 |  |
| 27 | B3Y-12 | 12 |  |
| 28 | B3Y-1 | 1 |  |
| 29 | B3Y-2 | 2 |  |
| 30 | B3Y-3 | 3 |  |
| 31 | B3Y-4 | 4 |  |
| 32 | B3Y-5 | 5 |  |
| 33 | B3Y-6 | 6 |  |
| 34 | B3Y-7 | 7 |  |
| 35 | B3Y-8 | 8 |  |
| 36 | B3Y-9 | 9 |  |
| 37 | 630Y-10 | 10 | 630 A039 (630) |
| 38 | 630Y-11 | 11 |  |
| 39 | 630Y-12 | 12 |  |
| 40 | 630Y-1 | 1 |  |
| 41 | 630Y-2 | 2 |  |
| 42 | 630Y-3 | 3 |  |
| 43 | 630Y-4 | 4 |  |
| 44 | 630Y-5 | 5 |  |
| 45 | 630Y-6 | 6 |  |
| 46 | 630Y-7 | 7 |  |
| 47 | 630Y-8 | 8 |  |
| 48 | 630Y-9 | 9 |  |
| 49 | Y3Y-10 | 10 | Y3 C102 (Y3) |
| 50 | Y3Y-11 | 11 |  |
| 51 | Y3Y-12 | 12 |  |
| 52 | Y3Y-1 | 1 |  |
| 53 | Y3Y-2 | 2 |  |
| 54 | Y3Y-3 | 3 |  |
| 55 | Y3Y-4 | 4 |  |
| 56 | Y3Y-5 | 5 |  |
| 57 | Y3Y-6 | 6 |  |
| 58 | Y3Y-7 | 7 |  |
| 59 | Y3Y-8 | 8 |  |
| 60 | Y3Y-9 | 9 |  |

Table S2 Precursor/Product ion pairs and parameters for MRM of compounds used in this study

| Compound | RT（min） | ion pairs (*m/z*) | Cone (V) | Collision (V) | Start (min) | End (min) |
| --- | --- | --- | --- | --- | --- | --- |
| Vitamin B_5_ | 6.45 | 220.0788＞72.1457 | 8 | 18 | 6.2 | 7 |
|  |  | 220.0788＞90.0621* |  | 14 |  |  |
| Vitamin B_1_ | 0.90 | 265.1364＞122.1015* | 28 | 14 | 0.7 | 1.3 |
|  |  | 265.1364＞144.0434 |  | 12 |  |  |
| NMN | 1.22 | 335.0885＞97.0161 | 28 | 26 | 1.0 | 1.8 |
|  |  | 335.0885＞123.0596* |  | 12 |  |  |
| Vitamin B_2_ | 7.80 | 377.1702＞172.0859 | 28 | 36 | 7.6 | 8.2 |
|  |  | 377.1702＞243.0935* |  | 22 |  |  |
| Folic acid | 7.43 | 442.1716＞176.0528 | 28 | 40 | 7.2 | 8.0 |
|  |  | 442.1716＞295.1421* |  | 20 |  |  |

*: Quantitative ion pairs

Table S3 Sequence of qPCR primers for RNA detection

| Gene | Upstream sequence (5’-3’) | Downstream sequence (5’-3’) |
| --- | --- | --- |
| *TNFα* | GCCTCCCTCTCATCAGTTCTA | GGCAGCCTTGTCCCTTG |
| *IL-1β* | GCAACTGTTCCTGAACTCAACT | ATCTTTTGGGGTCCGTCAACT |
| *IL-6* | CTGCAAGAGACTTCCATCCAG | AGTGGTATAGACAGGTCTGTTGG |
| *GAPDH* | TGAACGGGAAGCTCACTGG | GAGCTTCACAAAGTTGTCATTGAG |

Table S4 Identification list of secondary metabolites of *D. officinale*

| Classification | Numbers | t_R_ (min) | Identification | Formula | *m/z* | ppm | MS/MS Fragments |
| --- | --- | --- | --- | --- | --- | --- | --- |
| Phenols | A-1 | 2.44 | koaburaside | C_14_H_20_O_9_ | 331.1034 | -2.2 | 168.0433,153.0194,125.0242 |
|  | A-2 | 2.52 | Vanillic acid 4-β-D-glucoside | C_14_H_18_O_9_ | 329.0882 | 0.7 | 152.0115 |
|  | A-3 | 3.06 | Glucosyringic acid | C_15_H_20_O_10_ | 359.0984 | -0.8 | 197.0456,182.0219 |
|  | A-4 | 4.08 | darendoside A | C_19_H_28_O_11_ | 431.156 | -1.8 | 235.1174,99.0085 |
|  | A-5 | 4.24 | (2*S*)-1-*O*-(4-hydroxy-3-methoxybenzoyl)-3-(*O*-*α*-*D*-glucuronosyl)glycerol | C_17_H_22_O_12_ | 417.1034 | -2.5 | 152.0107 |
|  | A-6 | 4.42 | (7*S*,8*S*)-guaiacylglycerol 8-*O*-*β*-Dglucopyranoside | C_16_H_24_O_11_ | 391.1244 | -3.2 | 315.0712,153.0193 |
|  | A-7 | 4.56 | kelampayoside A | C_20_H_30_O_13_ | 477.1669 | -1.8 | 125.0242 |
|  | A-8 | 5.04 | icariside F2 | C_18_H_26_O_10_ | 401.1451 | -1.0 | 347.1138,269.1030,161.0462 |
|  | A-9 | 5.13 | lucidumoside C | C_27_H_36_O_14_ | 583.2036 | -1.0 | 419.171,329.1231 |
|  | A-10 | 5.27 | 1-*O*-[*α*-*L*-arabinofuranosyl(1→6)-*β*-D-glucopyranosyl]-erythro-1,2-bis(4-hydroxy-3-methoxyphenyl)propan | C_28_H_38_O_15_ | 613.214 | 0.0 | 567.2087,383.0772,195.0657 |
|  | A-11 | 5.35 | 4-(*β*-D-glucopyranosyloxy)-3,5-dimethoxyphenyl 6-*O*-(4-hydroxy-3-methoxybenzoyl)-*β*-D-glucopyranoside | C_28_H_36_O_17_ | 644.187 | -1.4 | 597.2176,481.1335,341.0861 |
|  | A-12 | 5.58 | 3-methoxybenzoic acid methyl ester 4-*O*-*α*-L-rhamnopyranosyl-(1''->2')-*β*-D-allopyranoside | C_21_H_30_O_13_ | 489.1608 | 1.5 | 327.1084,236.1038,165.0560 |
|  | A-13 | 5.85 | periclymenoside | C_33_H_44_O_18_ | 727.2459 | 0.8 | 501.1777,339.1235,179.0540 |
|  | A-14 | 6.02 | dilatioside B | C_34_H_46_O_19_ | 757.2564 | 0.1 | 531.1870,369.1351,339.1241 |
|  | A-15 | 7.03 | 5-*O*-caffeoyl-4-*O*-syringoylquinic acid | C_25_H_26_O_13_ | 533.1304 | -0.2 | 431.0972,283.0617,165.0552 |
|  | A-16 | 7.39 | lyoniresinol 9′*α*-*O*-*β*-D-xylopyranoside | C_27_H_36_O_12_ | 551.2137 | -0.8 | 359.1500,344.1260,208.0742 |
|  | A-17 | 9.12 | geoside | C_21_H_30_O_11_ | 457.1717 | 0.8 | 163.0767,148.0530 |
|  | A-18 | 9.37 | paeonolide | C_20_H_28_O_12_ | 459.151 | 0.8 | 327.1096,165.0558,164.0471 |
|  | A-19 | 9.73 | alangisesquin A | C_37_H_46_O_16_ | 745.2716 | 1.5 | 697.2714,535.1975,433.1513 |
|  | A-20 | 13.43 | 10*a*,14*b*-methylene-ditritin | C_31_H_32_O_8_ | 531.2025 | 0.7 | 391.1554,257.0805 |
| Flavonoids | B-1 | 5.30 | vicenin-2 | C_27_H_30_O_15_ | 593.1514 | -0.1 | 473.1088, 353.0667，163.0400 |
|  | B-2 | 5.75 | neoisoschaftoside | C_26_H_28_O_14_ | 563.1405 | -0.8 | 383.0768,353.0658 |
|  | B-3 | 5.95 | schaftoside | C_26_H_28_O_14_ | 563.1405 | -0.4 | 383.0781,353.0674 |
|  | B-4 | 6.38 | isoschaftoside | C_26_H_28_O_14_ | 563.1408 | -0.6 | 353.0670,325.0704 |
|  | B-5 | 6.46 | glucosyl-vitexin | C_27_H_30_O_15_ | 593.1515 | -1.0 | 413.0882,293.0456 |
|  | B-6 | 6.95 | rutin | C_27_H_30_O_16_ | 609.1463 | 0.1 | 300.0276,151.0039 |
|  | B-7 | 7.90 | apigenin 6-C-alpha-L-arabinopyranosyl-8-C-beta-D-xylopyranoside | C_25_H_26_O_13_ | 533.1302 | -0.7 | 443.0984,353.0674,325.0725 |
|  | B-8 | 8.59 | ambocin | C_26_H_28_O_14_ | 563.1403 | -0.6 | 383.0764,293.0457 |
|  | B-9 | 8.79 | apioside | C_26_H_28_O_14_ | 563.1408 | 0.6 | 383.0768,293.0457 |
|  | B-10 | 11.75 | naringenin | C_15_H_12_O_5_ | 271.061 | 0.3 | 151.0032,119.0500 |
| Lignan | C-1 | 6.18 | 5,5'-dimethoxylariciresinol 4-*O*-*β*-D-glucopyranoside | C_28_H_38_O_13_ | 581.2239 | -0.8 | 516.1298,179.0553,113.0246 |
|  | C-2 | 6.22 | liriodendrin | C_34_H_46_O_18_ | 787.2671 | -0.6 | 579.2094,417.1555,181.0505 |
|  | C-3 | 7.31 | hydroxysyringaresinol 9-*O*-*β*-D-glucopyranoside | C_28_H_36_O_14_ | 595.2034 | 0.0 | 357.1352 |
|  | C-4 | 8.72 | acanthoside B | C_28_H_36_O_13_ | 579.2084 | 0.3 | 417.1558,402.1321,181.0510 |
|  | C-5 | 9.91 | Reaxys ID 32411403 | C_37_H_46_O_16_ | 745.2716 | 1.0 | 643.2400,595.2175,367.1032, |
|  | C-6 | 13.11 | dendrocandin U | C_26_H_28_O_8_ | 468.2089 | 0.3 | 209.0820,151.0404,137.0253 |
| Glycolipids | D-1 | 16.91 | 1-*O*-linoloyl-3-*O*-*β*-D-galactopyranosyl-sn-glycerol | C_27_H_48_O_9_ | 561.3282 | -0.9 | 281.2482,279.2326 |
|  | D-2 | 15.09 | 3-{[6-*O*-(beta-D-Galactopyranosyl)-beta-D-galactopyranosyl]oxy}-2-hydroxypropyl (12*Z*,15*Z*)-12,15-octadecadienoate | C_33_H_58_O_14_ | 723.3812 | 3.3 | 397.1349,281.2483 |
|  | D-3 | 15.39 | gingerglycolipid B | C_33_H_58_O_14_ | 723.3813 | -0.1 | 415.1443,397.1345 |
|  | D-4 | 15.84 | 3-*O*-*β*-D-galactopyranosyl-1-*O*-[(9*Z*,12*Z*,15*Z*)-octadeca-9,12,15-trienoyl]-sn-glycerol | C_27_H_46_O_9_ | 559.3121 | -1.3 | 480.3078,277.2171,253.0923 |
|  | D-5 | 16.58 | 3-(beta-D-Galactopyranosyloxy)-2-hydroxypropyl (9*Z*,12*Z*)-9,12-octadecadienoate | C_27_H_48_O_9_ | 566.374 | -1.5 | 353.1998,279.2329 |
| Terpenes | E-1 | 2.95 | eleganoside-C | C_18_H_28_O_12_ | 435.1515 | 0.1 | 315.0717,152.0116,109.0292 |
|  | E-2 | 4.32 | sweroside | C_16_H_22_O_9_ | 357.1187 | -0.5 | 162.0304 |
|  | E-3 | 4.65 | geniposide | C_17_H_24_O_10_ | 387.1299 | 0.5 | 207.0670,192.0392,177.0190 |
|  | E-4 | 4.92 | yadanzioside D | C_29_H_40_O_16_ | 643.2252 | -1.7 | 405.1552,357.1335,266.0117 |
|  | E-5 | 6.07 | genipin 1-O-β-D-gentiobioside | C_23_H_34_O_15_ | 549.1816 | -1.0 | 323.0970,195.0659,179.0717, |
|  | E-6 | 6.09 | asaolaside | C_28_H_38_O_15_ | 613.214 | -0.2 | 357.1340,195.0659,179.0717, |
|  | E-7 | 6.17 | hedyoiridoidside B | C_22_H_38_O_13_ | 509.2242 | 1.1 | 463.2187,149.0463,89.0243 |
|  | E-8 | 8.93 | 7-*O*-acetyllaciniatoside IV | C_29_H_40_O_15_ | 627.2293 | 0.2 | 209.0824 |
|  | E-9 | 10.77 | 4-hydroxy-3,7-dimethyl-2,6-octadienyl-?-D-glucopyranosyl(1→3)-?-D-glucopyranoside | C_22_H_38_O_12_ | 493.2292 | 0.4 | 447.2246 |
|  | E-10 | 11.04 | acuminoside | C_21_H_36_O_10_ | 447.2238 | -0.3 | 315.1809,101.0247 |
| phospholipid | F-1 | 15.59 | 1-(9*Z*,12*Z*)-octadecadienoyl-2-hydroxy-sn-glycero-3-phosphocholine | C_26_H_50_NO_7_P | 564.3301 | -0.3 | 504.3094,279.2330,277.2172 |
|  | F-2 | 16.75 | 1-oleoyl-3-lysophosphatidylcholine | C_26_H_52_NO_7_P | 566.3462 | -1.0 | 281.2483 |
| Anthraquinone | G | 6.73 | ruberythric acid | C_25_H_26_O_13_ | 533.1304 | -0.4 | 443.0984,353.0663,325.0706 |
| Organic acid | H | 4.24 | γ-methyl-δ-hydroxy-pentanoic acid *β*-D-glucopyranoside | C_12_H_22_O_8_ | 293.124 | 0.3 | 182.0574,99.0086 |
| Oligosaccharide | I | 5.59 | shimaurinoside B | C_16_H_30_O_10_ | 381.1765 | -0.9 | 165.056 |

Table S5 Relative content of secondary metabolites in leaves of *D. officinale* (n = 2)

| Varity | Month | A-1 | A-2 | A-3 | A-4 | A-5 | A-6 | A-7 | A-8 | A9 | A-10 | A-11 | A-12 | A13 | A-14 |
| --- | --- | --- | --- | --- | --- | --- | --- | --- | --- | --- | --- | --- | --- | --- | --- |
| Y2 | 10 | 5.316 | 1.013 | 4.405 | 7.032 | 55.986 | - | 4.358 | 136.998 | 0.508 | 0.453 | - | 7.475 | - | 0.650 |
| Y2 | 11 | 5.767 | 1.334 | 3.245 | 2.992 | 13.955 | - | 4.666 | 45.328 | 1.249 | 0.842 | - | 2.476 | 0.489 | 0.564 |
| Y2 | 12 | 1.632 | 1.055 | 2.191 | 2.477 | 13.234 | - | 2.822 | 45.062 | 0.715 | - | - | 1.973 | - | - |
| Y2 | 1 | 0.835 | 1.030 | 1.802 | 2.463 | - | - | 2.642 | 35.204 | - | - | - | 1.843 | - | - |
| Y2 | 2 | 2.208 | 0.806 | 1.354 | 2.735 | 11.816 | - | 1.801 | 36.512 | - | - | - | 1.756 | - | - |
| Y2 | 3 | 2.201 | - | 1.554 | 2.451 | 6.926 | - | 2.006 | 23.040 | - | - | - | 1.902 | - | - |
| Y2 | 4 | 2.252 | - | 1.791 | 2.740 | 9.111 | - | 1.973 | 32.612 | - | - | - | 3.001 | - | - |
| Y2 | 5 | 1.784 | - | 1.589 | 2.560 | 9.812 | - | 1.474 | 32.195 | - | - | - | 2.643 | - | - |
| Y2 | 6 | 4.131 | 0.944 | 2.313 | 2.567 | 10.550 | - | 1.901 | 30.631 | 0.851 | - | - | 3.669 | - | - |
| Y2 | 7 | 4.493 | 1.442 | 2.754 | 1.012 | 38.060 | - | 1.182 | 34.482 | - | - | - | 0.455 | - | - |
| Y2 | 8 | 3.945 | - | 3.435 | 4.386 | 17.750 | - | 2.491 | 60.358 | - | - | - | - | - | - |
| Y2 | 9 | 2.330 | - | 2.300 | 3.839 | - | - | 1.754 | 44.135 | - | - | - | - | - | - |
| B1 | 10 | 3.045 | 7.409 | 4.881 | 3.049 | 46.234 | - | 1.971 | 59.993 | - | - | - | 4.389 | - | - |
| B1 | 11 | 2.459 | 5.304 | 2.974 | 2.340 | 21.539 | - | 1.105 | 32.805 | - | - | - | 2.026 | - | - |
| B1 | 12 | 1.151 | 5.031 | 3.143 | 2.844 | 23.673 | - | 1.084 | 39.524 | - | - | - | 1.958 | - | - |
| B1 | 1 | 0.686 | 5.010 | 2.426 | 2.484 | 22.832 | - | 0.996 | 33.943 | - | - | - | 1.935 | - | - |
| B1 | 2 | 1.989 | 3.826 | 2.194 | 2.619 | 20.813 | - | 0.793 | 30.497 | - | - | - | 1.702 | - | - |
| B1 | 3 | 2.425 | 3.372 | 2.631 | 4.008 | 26.686 | - | 0.882 | 33.185 | - | - | - | 2.178 | - | - |
| B1 | 4 | 2.206 | 3.420 | 3.262 | 4.396 | 35.967 | - | 1.142 | 44.359 | - | - | - | 3.760 | 6.008 | 0.458 |
| B1 | 5 | 1.130 | 2.763 | 2.653 | 3.425 | 29.115 | - | 0.821 | 39.846 | - | - | - | 4.385 | - | - |
| B1 | 6 | 3.914 | 3.650 | 4.423 | 4.877 | 27.074 | - | 1.333 | 37.297 | - | - | - | 4.174 | - | - |
| B1 | 7 | 4.419 | - | 7.247 | 2.119 | 38.136 | - | 1.509 | 5.306 | - | - | - | 0.648 | - | - |
| B1 | 8 | 4.970 | - | 6.331 | 4.230 | 37.400 | - | 1.548 | 6.061 | - | - | - | 1.060 | - | - |
| B1 | 9 | 2.252 | 2.523 | 4.141 | 2.209 | - | - | 0.876 | 43.018 | - | - | - | - | - | - |
| B3 | 10 | 3.048 | 1.120 | 2.026 | 2.109 | 18.193 | - | 1.145 | 54.009 | - | - | - | 3.449 | - | - |
| B3 | 11 | 2.211 | 1.083 | 1.409 | 3.258 | 9.940 | - | 3.655 | 50.538 | - | - | - | 2.071 | - | - |
| B3 | 12 | 1.118 | 1.192 | 1.659 | 4.924 | 12.166 | - | 4.444 | 68.948 | - | - | - | 2.485 | - | - |
| B3 | 1 | 0.731 | 0.972 | 1.225 | 3.985 | 12.972 | - | 3.751 | 55.893 | - | - | - | 2.468 | - | - |
| B3 | 2 | 1.573 | 0.944 | 1.012 | 4.086 | 15.414 | - | 3.307 | 55.512 | - | - | - | 2.065 | - | - |
| B3 | 3 | 2.002 | 0.378 | 1.405 | 4.741 | 20.580 | - | 2.797 | 48.758 | - | - | - | 3.053 | - | - |
| B3 | 4 | 1.345 | - | 1.023 | 4.057 | 12.231 | - | 2.492 | 45.637 | - | - | - | 2.434 | - | - |
| B3 | 5 | 1.065 | -0 | 0.900 | 3.120 | 11.749 | - | 2.256 | 46.684 | - | - | - | 2.679 | - | - |
| B3 | 6 | 7.081 | 2.319 | 3.071 | 5.531 | 18.773 | - | 4.265 | 65.744 | - | - | - | 4.849 | 0.433 | 0.121 |
| B3 | 7 | 4.167 | 1.022 | 3.436 | 6.086 | 20.941 | - | 5.275 | 5.143 | - | - | - | - | 0.128 | - |
| B3 | 8 | 5.695 | 1.082 | 4.220 | 6.788 | 21.249 | - | 5.515 | 92.304 | - | - | - | - | - | - |
| B3 | 9 | 1.075 | 1.354 | 1.534 | 1.678 | - | - | 2.431 | 32.475 | - | - | - | 1.801 | - | - |
| 630 | 10 | 2.981 | 1.003 | 2.535 | 1.788 | 23.343 | - | 2.182 | 48.914 | - | - | - | 3.700 | - | - |
| 630 | 11 | 2.216 | 0.653 | 1.171 | 1.192 | 9.779 | - | 1.117 | 22.581 | - | - | - | 1.796 | - | - |
| 630 | 12 | 2.241 | 1.109 | 2.339 | 3.268 | 17.860 | - | 2.395 | 47.050 | - | - | - | 3.553 | - | - |
| 630 | 1 | 0.851 | 0.866 | 1.493 | 2.348 | 16.946 | - | 1.732 | 35.721 | - | - | - | 2.859 | - | - |
| 630 | 2 | 2.506 | 0.810 | 1.364 | 2.675 | 17.366 | - | 1.557 | 32.372 | - | - | - | 1.840 | - | - |
| 630 | 3 | 1.327 | - | 0.827 | 2.736 | 10.819 | - | 1.562 | 30.708 | - | - | - | 1.747 | - | - |
| 630 | 4 | 1.890 | - | 1.394 | 3.053 | 13.156 | - | 1.194 | 29.041 | - | - | - | 2.716 | - | - |
| 630 | 5 | 1.477 | - | 1.898 | 4.282 | 15.269 | - | 1.358 | 34.634 | - | - | - | 5.073 | - | - |
| 630 | 6 | 5.050 | 0.986 | 2.883 | 5.486 | 21.882 | - | 1.682 | 43.193 | - | - | - | 5.964 | - | - |
| 630 | 7 | 5.066 | - | 4.158 | 8.063 | 24.735 | - | 2.420 | 4.550 | - | - | - | - | - | - |
| 630 | 8 | 4.670 | 0.859 | 3.635 | 7.125 | 16.815 | - | 2.366 | 42.779 | - | - | - | - | - | - |
| 630 | 9 | 0.875 | 0.976 | 1.687 | 0.974 | - | - | 0.837 | 18.754 | - | - | - | - | - | - |
| Y3 | 10 | 3.412 | 1.472 | 3.097 | 5.318 | 18.754 | - | 6.235 | 106.613 | 0.641 | - | - | 7.493 | - | - |
| Y3 | 11 | 3.708 | 1.887 | 2.093 | 2.423 | 32.560 | - | 1.549 | 56.866 | - | - | 0.493 | 2.596 | - | - |
| Y3 | 12 | 2.461 | 2.298 | 3.136 | 3.473 | 28.996 | - | 1.420 | 58.692 | 0.659 | - | - | 1.966 | - | - |
| Y3 | 1 | 0.994 | 1.378 | 1.424 | 1.870 | 21.305 | - | 0.975 | 42.824 | - | - | - | 1.657 | - | - |
| Y3 | 2 | 3.114 | 1.592 | 1.259 | 2.423 | - | - | 0.985 | 38.238 | - | - | - | 1.416 | - | - |
| Y3 | 3 | 1.920 | 0.812 | 1.472 | 3.825 | 25.303 | - | 0.855 | 39.365 | - | - | - | 2.335 | - | - |
| Y3 | 4 | 2.356 | 0.971 | 1.775 | 4.327 | 27.362 | - | 1.388 | 41.586 | - | - | - | 2.318 | - | - |
| Y3 | 5 | 1.737 | 1.060 | 1.695 | 2.939 | 30.400 | - | 1.100 | 50.445 | - | - | - | 3.736 | 77.580 | 0.437 |
| Y3 | 6 | 4.251 | 1.598 | 3.023 | 3.550 | 38.277 | - | 1.561 | 54.070 | - | - | - | 5.785 | - | - |
| Y3 | 7 | 4.105 | - | 3.196 | 5.454 | 15.407 | - | 2.492 | 49.770 | - | - | - | - | - | - |
| Y3 | 8 | 4.730 | - | 2.834 | 1.820 | 26.531 | - | 1.712 | 66.286 | - | - | - | - | - | - |
| Y3 | 9 | 2.962 | - | 2.034 | 1.174 | - | - | 0.767 | 47.218 | - | - | - | - | - | - |
| Type | Month | A-15 | A-16 | A-17 | A-18 | A-19 | A-20 | B-1 | B-2 | B-3 | B-4 | B-5 | B-6 | B-7 | B-8 |
| Y2 | 10 | 56.393 | 1.052 | 3.638 | 5.486 | 0.712 | - | 174.796 | 84.769 | 101.236 | 71.323 | 188.142 | 305.840 | 86.882 | 171.492 |
| Y2 | 11 | 149.444 | 1.306 | 2.466 | 5.083 | 0.886 | - | 78.864 | 59.787 | 115.957 | 51.047 | 111.921 | 545.529 | 164.299 | 189.063 |
| Y2 | 12 | 114.350 | 0.835 | 2.201 | 3.824 | - | - | 105.229 | 64.839 | 107.352 | 55.058 | 112.611 | 508.456 | 123.744 | 183.011 |
| Y2 | 1 | 103.934 | 0.727 | 1.496 | 3.514 | - | - | 88.315 | 52.900 | 101.975 | 52.783 | 97.400 | 428.866 | 109.520 | 133.439 |
| Y2 | 2 | 68.524 | 0.549 | 1.632 | 3.032 | - | - | 95.244 | 55.860 | 77.126 | 48.704 | 89.921 | 330.941 | 77.129 | 85.670 |
| Y2 | 3 | 66.157 | - | 1.022 | 3.332 | - | - | 44.092 | 35.171 | 73.222 | 14.075 | 3.060 | 31.800 | 4.360 | 2.025 |
| Y2 | 4 | 97.880 | - | 1.316 | 4.460 | - | 0.991 | 56.053 | 47.127 | 99.755 | 38.932 | 124.382 | 581.497 | 150.615 | 129.459 |
| Y2 | 5 | 70.976 | - | 1.265 | 3.758 | - | 0.833 | 64.606 | 42.987 | 73.412 | 38.377 | 123.434 | 426.709 | 100.435 | 85.808 |
| Y2 | 6 | 76.419 | - | 1.586 | 4.868 | - | 1.081 | 77.068 | 53.302 | 96.075 | 46.558 | 204.401 | 509.495 | 110.426 | 95.099 |
| Y2 | 7 | 2.282 | 0.483 | 0.484 | 6.763 | - | 0.701 | 0.968 | 5.890 | 231.870 | 10.937 | 2.340 | 264.486 | 3.489 | 63.268 |
| Y2 | 8 | 61.097 | - | - | 5.667 | - | - | 1.298 | 5.080 | 117.658 | 7.205 | 3.986 | 492.704 | 79.411 | 105.572 |
| Y2 | 9 | 69.853 | - | - | - | - | - | 109.488 | 71.609 | 71.609 | 11.562 | 2.701 | 31.584 | 4.705 | 1.237 |
| B1 | 10 | 2.234 | 0.851 | 2.001 | 13.422 | - | 0.607 | 14.415 | 5.680 | 10.916 | 2.255 | 171.798 | 52.513 | 1.875 | 117.424 |
| B1 | 11 | 1.362 | 0.507 | 1.047 | 7.772 | - | - | 10.182 | 3.495 | 5.573 | 3.123 | 124.905 | 45.661 | 1.060 | 81.701 |
| B1 | 12 | 1.657 | 0.822 | 1.483 | 11.198 | - | - | 9.730 | 7.969 | 11.220 | 1.234 | 179.111 | 93.735 | 2.135 | 114.027 |
| B1 | 1 | 1.365 | 0.675 | 1.188 | 8.827 | - | - | 8.429 | 4.288 | 7.273 | 0.897 | 251.345 | 73.682 | 1.249 | 86.658 |
| B1 | 2 | 0.989 | 0.523 | 0.927 | 7.075 | - | - | 6.391 | 1.776 | 4.719 | 0.717 | 104.814 | 54.663 | 0.753 | 65.192 |
| B1 | 3 | 0.608 | - | 5.977 | 5.552 | - | 0.482 | 8.319 | 5.273 | 10.325 | 7.117 | 1.065 | 4.212 | - | 48.524 |
| B1 | 4 | 0.966 | 1.167 | 7.530 | 6.744 | - | 0.566 | 10.053 | 4.951 | 5.358 | 144.762 | 147.069 | 54.691 | 44.616 | 109.848 |
| B1 | 5 | 3.764 | - | 1.194 | 12.267 | - | 1.526 | 8.026 | 2.346 | 5.499 | - | 234.721 | 54.329 | 1.037 | 68.863 |
| B1 | 6 | 24.524 | - | 1.572 | 13.350 | - | 1.181 | 13.625 | 4.885 | 27.384 | 4.395 | 223.430 | 105.664 | 6.756 | 132.587 |
| B1 | 7 | 4.439 | 0.509 | 1.048 | 15.468 | - | 0.203 | - | - | 7.103 | 0.695 | 1.953 | 117.701 | 5.504 | 142.225 |
| B1 | 8 | 10.620 | - | 0.968 | 16.613 | - | - | - | - | 8.364 | - | 1.976 | 169.148 | 15.207 | 226.590 |
| B1 | 9 | 10.463 | - | - | - | - | - | 10.826 | 6.394 | 6.394 | 7.862 | 1.646 | 4.903 | - | - |
| B3 | 10 | 5.277 | - | 1.674 | 3.870 | - | - | 209.304 | 179.544 | 94.851 | 157.907 | 82.581 | 110.203 | 5.423 | 26.968 |
| B3 | 11 | 0.735 | - | 0.974 | 4.105 | - | - | 164.805 | 152.330 | 86.102 | 129.446 | 4.197 | 179.563 | 0.736 | - |
| B3 | 12 | 1.129 | - | 1.337 | 5.579 | - | - | 204.230 | 195.149 | 120.377 | 176.036 | 5.765 | 243.577 | 1.000 | - |
| B3 | 1 | 0.935 | - | 0.921 | 4.821 | - | - | 188.389 | 154.487 | 107.740 | 157.564 | 0.802 | 250.536 | 0.758 | - |
| B3 | 2 | 0.614 | - | 0.743 | 3.976 | - | - | 150.290 | 146.241 | 90.886 | 133.933 | 2.174 | 123.857 | 0.659 | - |
| B3 | 3 | 3.863 | - | 1.924 | 7.506 | - | 1.211 | 97.082 | 86.813 | 58.139 | 14.125 | 3.790 | 18.463 | - | - |
| B3 | 4 | - | - | - | 4.710 | - | - | 135.015 | 129.448 | 77.563 | 116.411 | 3.595 | 252.260 | - | 1.478 |
| B3 | 5 | - | - | - | 4.412 | - | - | 117.499 | 120.604 | 72.267 | 106.751 | 2.968 | 168.650 | 0.378 | 1.020 |
| B3 | 6 | 2.484 | - | 1.397 | 8.508 | - | - | 209.958 | 195.818 | 157.677 | 183.064 | 21.470 | 256.286 | 2.102 | 7.058 |
| B3 | 7 | 0.770 | - | - | 10.102 | - | - | 1.185 | 4.543 | 177.960 | 9.908 | 5.923 | 326.638 | 1.155 | 17.195 |
| B3 | 8 | - | - | - | 9.015 | - | - | 1.184 | 4.018 | 177.692 | 8.055 | 5.379 | 277.250 | 0.794 | 26.090 |
| B3 | 9 | 0.935 | - | - | - | - | - | 267.259 | 234.070 | 234.070 | 14.617 | 5.181 | 5.792 | - | - |
| 630 | 10 | 11.924 | - | 3.821 | 8.650 | - | - | 6.951 | 5.453 | 9.138 | 4.563 | 74.946 | 189.330 | 10.848 | 190.891 |
| 630 | 11 | 7.169 | - | 2.092 | 4.685 | - | - | 4.066 | 2.879 | 5.455 | 2.690 | 45.712 | 144.290 | 6.396 | 123.216 |
| 630 | 12 | 13.793 | - | 4.032 | 9.839 | - | 0.729 | 7.334 | 5.261 | 10.011 | 5.263 | 74.569 | 281.142 | 12.448 | 202.664 |
| 630 | 1 | 10.304 | - | 2.590 | 7.406 | - | 0.733 | 5.708 | 4.032 | 7.288 | 3.785 | 49.664 | 264.503 | 8.369 | 146.388 |
| 630 | 2 | 7.813 | - | 2.255 | 5.889 | - | 0.626 | 4.734 | 3.445 | 6.603 | 3.493 | 54.531 | 199.720 | 7.432 | 138.126 |
| 630 | 3 | 2.539 | - | 1.208 | 4.676 | - | 1.027 | 51.043 | 46.559 | 29.784 | 7.999 | 2.078 | 10.799 | - | - |
| 630 | 4 | 5.738 | - | 2.389 | 7.568 | - | 3.266 | 4.363 | 4.259 | 6.478 | 2.897 | 64.216 | 299.622 | 9.185 | 115.599 |
| 630 | 5 | 9.141 | - | 3.054 | 10.865 | - | 3.175 | 6.191 | 6.167 | 8.628 | 4.831 | 118.418 | 332.653 | 10.371 | 165.555 |
| 630 | 6 | 26.513 | - | 3.595 | 13.660 | - | 2.488 | 19.149 | 16.738 | 32.054 | 17.418 | 104.544 | 360.524 | 35.150 | 142.593 |
| 630 | 7 | 22.302 | - | 0.715 | 12.428 | - | 0.802 | - | 1.168 | 29.661 | 4.632 | 2.714 | 478.303 | 29.570 | 248.958 |
| 630 | 8 | 35.210 | - | 0.857 | 8.245 | - | - | - | 2.196 | 52.052 | 2.889 | 2.870 | 430.400 | 47.425 | 232.315 |
| 630 | 9 | 6.195 | - | - | - | - | - | 10.529 | 7.140 | 7.140 | 11.360 | 2.403 | 17.341 | - | - |
| Y3 | 10 | 1.300 | - | 1.379 | 7.136 | - | - | 243.454 | 215.262 | 132.947 | 187.689 | 7.264 | 194.055 | 1.211 | 2.543 |
| Y3 | 11 | 1.624 | 0.756 | 2.828 | 4.936 | - | - | 242.175 | 228.298 | 137.326 | 215.952 | 103.846 | 115.868 | 1.646 | 11.106 |
| Y3 | 12 | 0.570 | 0.941 | 3.051 | 6.226 | - | - | 307.753 | 285.775 | 164.224 | 262.171 | 166.587 | 140.336 | 0.688 | 5.150 |
| Y3 | 1 | 2.277 | 0.546 | 1.541 | 3.412 | - | - | 196.528 | 163.712 | 92.700 | 144.280 | 102.010 | 82.462 | 2.238 | 8.445 |
| Y3 | 2 | 2.631 | 0.584 | 1.826 | 3.532 | - | - | 150.743 | 140.272 | 74.597 | 128.995 | 109.316 | 52.345 | 2.885 | 43.013 |
| Y3 | 3 | 11.508 | - | 2.067 | 5.017 | - | - | 238.381 | 202.656 | 126.617 | 14.112 | 2.742 | 8.633 | 0.751 | 0.738 |
| Y3 | 4 | 3.064 | - | 2.177 | 5.445 | - | - | 222.823 | 225.391 | 128.890 | 198.108 | 143.661 | 126.129 | 3.787 | 68.200 |
| Y3 | 5 | 4.433 | 7.273 | 4.195 | 2.557 | 0.513 | 0.601 | 265.432 | 222.780 | 63.296 | 186.630 | 49.003 | 85.856 | 38.141 | 73.126 |
| Y3 | 6 | 1.727 | - | 2.355 | 6.631 | - | 1.108 | 281.902 | 246.749 | 160.498 | 252.129 | 50.308 | 242.702 | 2.148 | 3.553 |
| Y3 | 7 | 130.272 | - | 0.728 | 5.513 | - | - | 0.863 | 6.350 | 77.977 | 13.609 | 4.919 | 575.940 | 170.491 | 171.602 |
| Y3 | 8 | 18.402 | - | - | 5.895 | -0 | - | 1.112 | 5.200 | 220.320 | 10.365 | 2.808 | 286.599 | 22.869 | 44.081 |
| Y3 | 9 | - | - | - | - | - | - | 261.081 | 224.811 | 224.811 | 16.713 | 1.445 | 10.449 | - | - |
| Type | Month | B-9 | B-10 | C-1 | C-2 | C-3 | C-4 | C-5 | C-6 | D-1 | D-2 | D-3 | D-4 | D-5 | E-1 |
| Y2 | 10 | 183.410 | 5.992 | 2.177 | 7.334 | 1.431 | 11.661 | 1.885 | 3.092 | 0.858 | 6.053 | 2.488 | 43.384 | 10.149 | 1.850 |
| Y2 | 11 | 192.544 | 4.693 | 0.656 | 2.986 | 2.208 | 8.107 | 2.677 | 4.027 | - | 0.981 | - | 9.600 | 1.947 | 0.658 |
| Y2 | 12 | 196.042 | 0.979 | - | 2.469 | 0.624 | 4.789 | 1.508 | 2.272 | 3.024 | 1.235 | 4.800 | 212.448 | 3.461 | - |
| Y2 | 1 | 154.003 | 0.634 | - | 2.425 | - | 3.948 | 1.092 | 1.437 | 2.798 | 0.702 | 3.458 | 118.564 | 2.166 | - |
| Y2 | 2 | 106.429 | 1.053 | - | 2.243 | - | 4.769 | 0.827 | 1.897 | 1.311 | 0.681 | 2.089 | 77.707 | 1.765 | 0.496 |
| Y2 | 3 | 111.444 | 0.919 | - | 1.958 | - | 3.720 | - | 3.724 | 4.084 | 1.200 | 3.016 | 142.696 | 6.583 | - |
| Y2 | 4 | 179.520 | 1.355 | - | 3.241 | - | 5.164 | - | 4.658 | 4.512 | 1.580 | 4.656 | 220.059 | 10.648 | - |
| Y2 | 5 | 122.669 | 1.695 | - | 2.670 | - | 4.005 | - | 3.597 | 5.793 | 1.187 | 3.305 | 284.683 | 18.763 | - |
| Y2 | 6 | 122.133 | 2.785 | - | 3.154 | 1.187 | 5.832 | 1.198 | 5.652 | 8.200 | 3.318 | 4.329 | 82.010 | 15.228 | - |
| Y2 | 7 | 47.737 | 3.744 | 0.855 | 4.508 | - | 8.198 | 0.543 | 8.098 | 10.325 | 1.168 | 1.859 | 215.783 | 40.694 | 0.662 |
| Y2 | 8 | 84.522 | 4.022 | 0.600 | 5.406 | - | 7.470 | - | 5.076 | 17.705 | 1.841 | 2.424 | 92.650 | 50.397 | 1.259 |
| Y2 | 9 | 132.989 | - | - | 3.656 | - | 4.361 | - | 3.344 | - | 6.115 | 12.297 | 135.803 | 55.253 | 0.824 |
| B1 | 10 | 158.277 | 2.093 | 0.924 | 2.803 | 0.638 | 7.621 | 0.717 | 4.849 | 0.458 | 0.582 | 3.522 | 220.704 | 20.260 | 0.640 |
| B1 | 11 | 100.122 | 0.808 | 0.507 | 1.707 | - | 5.053 | 0.440 | 2.270 | 3.319 | - | - | 133.918 | 5.184 | - |
| B1 | 12 | 140.802 | 0.741 | 0.724 | 3.263 | - | 7.940 | 0.971 | 2.714 | 0.736 | 0.553 | 2.128 | 186.961 | 5.827 | - |
| B1 | 1 | 110.496 | 0.556 | 0.581 | 3.368 | - | 6.696 | 0.730 | 2.385 | 2.129 | 0.559 | 2.855 | 185.635 | 4.437 | - |
| B1 | 2 | 82.749 | 0.650 | 0.488 | 2.510 | - | 6.129 | 0.517 | 2.441 | 2.803 | 0.512 | 1.437 | 142.998 | 3.737 | - |
| B1 | 3 | 52.706 | 3.189 | 3.301 | 6.608 | - | 4.109 | 1.671 | 2.573 | 29.293 | 0.387 | 70.206 | 118.257 | 12.538 | - |
| B1 | 4 | 67.956 | 3.236 | 4.522 | 2.739 | - | 4.606 | 1.988 | 3.515 | 40.005 | - | 71.478 | 125.884 | 14.448 | - |
| B1 | 5 | 120.667 | 1.574 | - | 3.469 | - | 6.748 | - | 8.373 | 2.397 | - | 2.870 | 205.453 | 19.718 | - |
| B1 | 6 | 175.715 | 2.837 | - | 3.705 | - | 8.478 | - | 7.615 | 9.587 | - | - | 113.748 | 26.119 | - |
| B1 | 7 | 95.802 | 2.296 | 0.984 | 5.809 | - | 11.776 | - | 10.128 | 2.380 | 3.163 | 6.901 | 113.194 | 28.654 | 0.782 |
| B1 | 8 | 185.887 | 3.911 | 0.964 | 6.897 | - | 11.370 | - | 8.331 | 2.511 | 1.089 | 1.545 | 86.633 | 63.836 | 0.843 |
| B1 | 9 | 125.325 | - | - | 4.328 | - | 6.826 | - | 5.951 | - | 3.086 | 8.424 | 120.317 | 53.272 | - |
| B3 | 10 | 36.315 | 4.641 | 0.510 | 2.651 | - | 4.708 | - | 5.203 | 1.091 | - | - | 118.646 | 6.943 | 0.534 |
| B3 | 11 | - | 0.734 | - | 1.139 | - | 2.663 | 0.673 | 2.166 | 2.119 | - | - | 143.821 | 2.771 | - |
| B3 | 12 | - | 0.777 | - | 2.095 | 0.681 | 4.450 | 1.548 | 3.272 | 1.104 | - | 1.357 | 142.068 | 2.690 | - |
| B3 | 1 | - | - | - | 2.355 | - | 4.460 | 1.027 | 3.038 | 2.895 | - | 1.093 | 82.634 | 1.236 | - |
| B3 | 2 | - | - | - | 2.006 | - | 4.058 | 0.771 | 2.704 | 2.076 | - | 0.627 | 103.062 | 1.594 | - |
| B3 | 3 | 67.381 | 1.181 | - | 3.091 | - | 6.220 | - | 5.402 | 7.451 | - | 0.785 | 175.511 | 12.448 | - |
| B3 | 4 | 2.227 | - | - | 2.081 | - | 3.602 | - | 4.065 | 4.018 | - | 1.078 | 153.304 | 6.721 | - |
| B3 | 5 | 1.436 | 1.284 | - | 1.851 | - | 3.140 | - | 4.639 | 4.761 | - | - | 270.537 | 11.520 | - |
| B3 | 6 | 10.857 | 2.383 | 0.856 | 2.806 | 1.232 | 5.565 | 1.391 | 7.407 | 8.173 | - | - | 117.791 | 21.496 | - |
| B3 | 7 | 12.561 | 1.574 | 0.927 | 4.715 | - | 6.828 | 0.573 | 7.425 | 11.371 | 3.622 | 4.490 | 176.559 | 52.481 | 0.730 |
| B3 | 8 | 18.170 | 2.043 | 0.801 | 5.079 | - | 7.943 | - | 7.102 | 6.457 | - | 0.737 | 75.906 | 34.461 | 0.816 |
| B3 | 9 | 8.622 | - | - | 1.148 | - | 1.676 | - | 1.083 | 0.781 | - | 3.213 | 159.882 | 9.063 | - |
| 630 | 10 | 225.231 | 2.872 | - | 3.575 | 0.681 | 5.672 | 0.682 | 3.835 | 0.468 | 0.525 | 3.178 | 241.775 | 16.567 | 0.645 |
| 630 | 11 | 152.127 | 0.990 | - | 2.184 | - | 4.056 | - | 3.151 | 2.200 | - | - | 106.843 | 3.797 | - |
| 630 | 12 | 246.040 | 3.224 | 0.081 | 5.496 | 1.027 | 9.145 | 1.088 | 9.546 | 1.081 | - | 1.762 | 183.012 | 6.329 | 0.637 |
| 630 | 1 | 183.224 | 1.825 | 0.057 | 4.571 | 0.632 | 7.460 | 0.712 | 6.264 | 0.695 | - | 1.087 | 130.289 | 3.088 | - |
| 630 | 2 | 161.545 | 0.992 | 0.085 | 3.724 | 0.451 | 6.456 | 0.733 | 3.899 | 2.956 | 0.458 | 1.275 | 131.132 | 3.457 | 0.430 |
| 630 | 3 | 43.583 | 0.853 | - | 1.851 | - | 3.507 | - | 3.975 | 3.658 | - | - | 97.363 | 6.768 | - |
| 630 | 4 | 167.575 | 2.185 | - | 4.425 | - | 5.860 | - | 8.302 | 4.287 | - | - | 166.973 | 12.938 | - |
| 630 | 5 | 231.968 | 4.766 | - | 7.271 | - | 9.325 | - | 16.893 | 1.069 | 3.086 | 13.454 | 344.640 | 35.662 | - |
| 630 | 6 | 205.561 | 5.236 | - | 8.316 | - | 10.436 | 0.871 | 12.010 | 8.985 | - | - | 130.520 | 32.266 | - |
| 630 | 7 | 190.220 | 3.485 | - | 10.556 | - | 11.004 | - | 11.352 | 7.259 | 2.122 | 4.427 | 242.925 | 52.528 | 1.115 |
| 630 | 8 | 187.866 | 4.268 | - | 6.616 | - | 8.994 | - | 6.977 | 4.927 | 1.630 | 2.386 | 112.587 | 44.825 | 1.217 |
| 630 | 9 | 187.884 | - | - | 1.785 | - | 2.664 | - | - | - | 4.863 | 33.186 | 164.217 | 17.674 | - |
| Y3 | 10 | 3.325 | 1.918 | 0.706 | 3.492 | - | 4.937 | 0.763 | 3.653 | 0.985 | - | 0.884 | 144.598 | 14.162 | 0.800 |
| Y3 | 11 | 15.086 | 1.290 | 0.538 | 2.136 | 0.526 | 4.289 | 0.552 | 1.849 | 3.314 | - | - | 183.086 | 5.138 | - |
| Y3 | 12 | 6.727 | 0.931 | 0.933 | 2.054 | 0.940 | 4.975 | 1.300 | 1.814 | 1.537 | 1.159 | 4.538 | 186.785 | 4.555 | - |
| Y3 | 1 | 10.973 | 1.310 | - | 1.959 | 0.505 | 4.488 | - | 2.515 | 0.982 | - | 0.817 | 141.608 | 3.242 | - |
| Y3 | 2 | 50.865 | 0.871 | - | 1.479 | 0.495 | 3.938 | 0.649 | 1.800 | 1.675 | 0.575 | 1.697 | 76.779 | 2.210 | - |
| Y3 | 3 | 32.963 | 2.655 | 0.711 | 2.299 | - | 5.445 | - | 5.018 | 7.425 | - | 0.968 | 214.790 | 11.372 | - |
| Y3 | 4 | 90.003 | 1.616 | 0.842 | 2.336 | - | 5.044 | - | 3.324 | 7.237 | - | 1.791 | 260.887 | 11.242 | - |
| Y3 | 5 | 41.287 | 4.989 | 1.381 | 120.854 | - | 2.735 | 4.120 | 3.356 | 26.948 | 2.273 | 15.408 | 120.200 | 8.584 | - |
| Y3 | 6 | 4.843 | 5.323 | 0.916 | 4.045 | 0.789 | 7.907 | 0.777 | 8.644 | 8.528 | - | - | 135.584 | 36.542 | - |
| Y3 | 7 | 138.175 | 1.267 | 0.613 | 4.629 | - | 6.388 | - | 3.891 | 11.452 | 3.800 | 5.829 | 201.296 | 40.831 | 0.799 |
| Y3 | 8 | 32.327 | 3.108 | 0.704 | 5.534 | - | 6.297 | - | 3.641 | 9.031 | 0.820 | 1.136 | 81.249 | 47.855 | 0.843 |
| Y3 | 9 | - | - | - | 2.750 | - | 3.547 | - | 1.961 | - | 3.181 | 4.678 | 85.074 | 41.913 | - |
| Type | Month | E-2 | E-3 | E-4 | E-5 | E-6 | E-7 | E-8 | E-9 | E-10 | F-1 | F-2 | G | H | I |
| Y2 | 10 | 0.420 | 0.547 | 1.036 | 2.096 | 1.350 | 16.091 | 0.770 | 3.293 | 6.236 | 10.345 | 6.543 | 63.966 | 1.684 | 3.323 |
| Y2 | 11 | - | 0.432 | 0.860 | - | 2.489 | 18.364 | 1.009 | 17.565 | 4.631 | 2.394 | 1.398 | 117.577 | 0.889 | 2.235 |
| Y2 | 12 | - | - | - | - | 0.764 | 10.194 | - | 8.667 | 2.089 | 38.342 | 16.451 | 101.533 | 0.798 | 2.465 |
| Y2 | 1 | - | - | - | - | 0.674 | 10.342 | - | 6.227 | 0.948 | 22.669 | 13.486 | 96.152 | 0.661 | 2.131 |
| Y2 | 2 | - | - | - | - | 0.555 | 5.930 | - | 3.376 | 0.629 | 50.977 | 6.717 | 60.406 | 0.523 | 2.186 |
| Y2 | 3 | - | - | - | - | - | 8.357 | - | 3.398 | - | 115.794 | 23.350 | 5.031 | - | 2.150 |
| Y2 | 4 | - | - | - | - | - | 10.160 | - | 3.267 | 0.873 | 143.009 | 41.738 | 117.945 | - | 3.047 |
| Y2 | 5 | - | - | - | - | - | 6.616 | - | 5.058 | 0.901 | 164.640 | 75.874 | 83.007 | - | 4.087 |
| Y2 | 6 | - | - | - | - | 1.218 | 7.721 | - | 4.209 | 1.240 | 69.849 | 20.291 | 83.641 | - | 4.307 |
| Y2 | 7 | - | - | 0.594 | 1.613 | - | 2.427 | - | 1.990 | 0.867 | 48.666 | 107.129 | - | 0.581 | 7.638 |
| Y2 | 8 | - | 0.639 | - | 0.976 | - | 7.508 | - | 1.239 | - | 75.355 | 91.821 | - | 1.026 | 9.058 |
| Y2 | 9 | - | - | - | - | - | 9.181 | - | 1.239 | 3.119 | 3.553 | 90.792 | 4.576 | - | 4.741 |
| B1 | 10 | - | - | 0.745 | - | - | 9.783 | - | 8.114 | 6.254 | 4.058 | 91.362 | 0.518 | 2.177 | 4.605 |
| B1 | 11 | - | - | 0.495 | 0.773 | 0.516 | 5.741 | - | 5.232 | 2.459 | 56.644 | 24.053 | - | 1.186 | 2.485 |
| B1 | 12 | - | 0.503 | 0.662 | 0.736 | 0.698 | 7.316 | - | 7.758 | 2.927 | 9.626 | 28.454 | - | 1.352 | 2.906 |
| B1 | 1 | - | - | 0.671 | 0.694 | 0.581 | 5.595 | - | 5.568 | 1.836 | 15.814 | 29.800 | - | 1.204 | 2.775 |
| B1 | 2 | - | - | 0.514 | 0.508 | 0.531 | 5.040 | - | 4.019 | 1.234 | 60.483 | 17.899 | - | 1.115 | 2.040 |
| B1 | 3 | - | 0.809 | 0.683 | - | 0.450 | 4.361 | 0.551 | 2.545 | 1.582 | 172.223 | 24.730 | - | 1.576 | 3.440 |
| B1 | 4 | - | - | - | - | 0.521 | 6.235 | 0.668 | 2.934 | 1.273 | 195.122 | 29.878 | 0.362 | 1.937 | 5.131 |
| B1 | 5 | - | - | - | - | - | 7.795 | - | 4.311 | 1.572 | 40.270 | 73.647 | - | 1.795 | 5.447 |
| B1 | 6 | 0.859 | - | - | 0.996 | 1.106 | 9.459 | - | 6.932 | 1.481 | 79.796 | 43.678 | 5.489 | 1.699 | 6.228 |
| B1 | 7 | - | - | 0.742 | 2.111 | - | 11.702 | - | 2.603 | 0.773 | 7.212 | 82.272 | 0.539 | 2.502 | 6.790 |
| B1 | 8 | - | - | 0.987 | 2.549 | - | 13.413 | - | 2.506 | 0.911 | 9.963 | 81.647 | 0.921 | 2.597 | 6.969 |
| B1 | 9 | - | - | - | 1.528 | - | 7.566 | - | 2.712 | - | 1.343 | 85.797 | 0.754 | 1.406 | 4.331 |
| B3 | 10 | - | - | - | 1.152 | - | 2.217 | - | 3.535 | 2.439 | 25.354 | 24.259 | 3.789 | 0.568 | 5.661 |
| B3 | 11 | - | - | 0.245 | - | - | 8.849 | - | 5.907 | 2.587 | 40.032 | 12.362 | 0.570 | - | 3.794 |
| B3 | 12 | - | - | 0.637 | - | 0.689 | 11.081 | - | 8.146 | 3.642 | 9.104 | 11.512 | 0.773 | - | 5.045 |
| B3 | 1 | - | - | 0.629 | - | 0.592 | 8.486 | - | 5.684 | 2.652 | 25.173 | 5.793 | 0.689 | - | 4.431 |
| B3 | 2 | - | - | - | - | - | 8.446 | - | 4.648 | 1.848 | 49.132 | 7.107 | 0.583 | - | 3.442 |
| B3 | 3 | - | - | - | - | - | 7.709 | - | 3.504 | 2.196 | 111.665 | 36.516 | 0.754 | - | 4.954 |
| B3 | 4 | - | - | - | - | - | 6.712 | - | 2.385 | - | 91.761 | 23.512 | - | - | 4.429 |
| B3 | 5 | - | - | - | - | - | 5.990 | - | 2.820 | - | 126.448 | 47.805 | - | - | 4.747 |
| B3 | 6 | 2.058 | - | - | 0.499 | 0.958 | 9.744 | - | 6.440 | 0.931 | 78.487 | 32.042 | 1.763 | - | 6.930 |
| B3 | 7 | - | 0.663 | 0.673 | 0.940 | 0.250 | 15.866 | - | 2.275 | 0.865 | 51.731 | 93.696 | - | 0.964 | 9.120 |
| B3 | 8 | - | - | - | 1.089 | - | 12.447 | - | 2.761 | 1.102 | 36.060 | 50.294 | - | 1.016 | 7.955 |
| B3 | 9 | - | - | - | - | - | 7.638 | - | 10.588 | 1.562 | 2.584 | 43.174 | - | - | 3.616 |
| 630 | 10 | - | - | 0.610 | 1.436 | 0.398 | 8.712 | - | 5.242 | 3.776 | 7.606 | 79.571 | 7.251 | 1.064 | 4.278 |
| 630 | 11 | - | - | 0.469 | 0.871 | 0.457 | 4.911 | - | 2.924 | 2.119 | 44.227 | 16.413 | 4.988 | 0.499 | 1.876 |
| 630 | 12 | - | - | 1.025 | 1.434 | 0.877 | 9.044 | - | 5.600 | 2.088 | 25.007 | 23.093 | 9.737 | 0.898 | 3.524 |
| 630 | 1 | - | - | 0.824 | 1.196 | 0.578 | 5.961 | - | 2.946 | 0.885 | 8.512 | 16.248 | 7.031 | 0.655 | 2.943 |
| 630 | 2 | - | - | 0.616 | 0.629 | 0.508 | 5.648 | - | 3.085 | 0.630 | 83.130 | 13.880 | 5.253 | 0.577 | 2.310 |
| 630 | 3 | - | - | - | - | - | 4.627 | - | 2.121 | 1.231 | 63.189 | 20.777 | - | - | 1.662 |
| 630 | 4 | - | - | - | 0.876 | - | 4.450 | - | 1.497 | - | 104.002 | 35.318 | 5.279 | - | 3.799 |
| 630 | 5 | - | - | - | 1.587 | - | 6.680 | - | 2.470 | 1.081 | 13.156 | 121.849 | 8.304 | - | 5.481 |
| 630 | 6 | - | 0.934 | 1.125 | 1.994 | 0.815 | 6.731 | - | 2.412 | - | 80.732 | 49.260 | 28.780 | 1.600 | 6.921 |
| 630 | 7 | - | - | 0.836 | 2.796 | - | 8.061 | - | 1.584 | - | 27.856 | 165.803 | 1.652 | 3.223 | 7.263 |
| 630 | 8 | - | - | 0.777 | 2.178 | - | 7.607 | - | 2.975 | 0.755 | 27.156 | 72.361 | 2.328 | 1.311 | 6.194 |
| 630 | 9 | - | - | - | 1.186 | - | 4.895 | - | 5.869 | 0.748 | 0.762 | 92.308 | 0.590 | - | 3.208 |
| Y3 | 10 | - | - | 0.562 | 0.812 | - | 14.593 | - | 4.690 | 5.633 | 14.194 | 44.967 | 0.855 | 1.054 | 8.959 |
| Y3 | 11 | - | - | - | 0.687 | 0.564 | 1.800 | - | 9.159 | 1.967 | 65.904 | 21.729 | 1.326 | 0.464 | 3.744 |
| Y3 | 12 | - | - | 0.628 | 0.588 | 0.838 | 2.765 | - | 13.108 | 1.938 | 26.723 | 19.542 | 0.537 | 0.245 | 3.738 |
| Y3 | 1 | - | - | - | 0.608 | - | 2.365 | - | 5.995 | 1.120 | 26.139 | 17.319 | 2.028 | - | 3.418 |
| Y3 | 2 | - | - | - | 0.399 | 0.575 | 1.855 | - | 4.515 | 0.700 | 51.409 | 8.457 | 2.092 | - | 2.219 |
| Y3 | 3 | - | - | - | 0.767 | - | 2.660 | - | 3.408 | 2.321 | 134.681 | 37.558 | 0.953 | - | 4.259 |
| Y3 | 4 | - | - | - | - | - | 2.873 | - | 4.425 | - | 145.048 | 46.358 | 2.942 | - | 5.088 |
| Y3 | 5 | - | - | - | 0.401 | - | 2.828 | 1.166 | 4.121 | 2.051 | 150.644 | 20.509 | 4.425 | - | 5.878 |
| Y3 | 6 | - | - | 0.824 | 1.249 | 0.864 | 2.229 | - | 8.282 | 1.384 | 96.224 | 44.628 | 1.702 | - | 5.511 |
| Y3 | 7 | - | - | - | - | - | 11.902 | - | 2.831 | - | 46.017 | 128.393 | 10.860 | 0.802 | 7.629 |
| Y3 | 8 | - | - | - | 1.304 | - | 4.532 | - | 2.384 | 0.873 | 43.215 | 78.016 | 1.223 | - | 8.192 |
| Y3 | 9 | - | - | - | 0.792 | - | 2.608 | - | 1.462 | 1.328 | 3.394 | 54.157 | - | - | 3.249 |

Table S6 Linear regression data, precision, and repeatability for 17 amino acids

| Compound | Regression equation | R^2^ | Linear range  （mg·g^-1^） | Precision RSD% | Repeatability RSD% |
| --- | --- | --- | --- | --- | --- |
| Asp | Y=0.0002X-4.8609 | 0.997 | 5-125 | 0.35 | 1.50 |
| Ser | Y=0.0002X-4.6665 | 0.997 | 5-125 | 0.50 | 3.98 |
| Glu | Y=0.0002X-4.2853 | 0.997 | 5-125 | 0.20 | 1.32 |
| Gly | Y=0.0002X-6.3015 | 0.993 | 5-125 | 0.65 | 3.03 |
| His | Y=0.0002X-3.6467 | 0.998 | 5-125 | 0.49 | 4.91 |
| Arg | Y=0.0002X-10.256 | 0.997 | 5-125 | 1.86 | 2.73 |
| Thr | Y=0.0002X-13.298 | 0.997 | 5-125 | 1.51 | 2.14 |
| Ala | Y=0.0002X-4.8044 | 0.996 | 5-125 | 0.38 | 1.88 |
| Pro | Y=0.0002X-3.2738 | 0.996 | 5-125 | 0.31 | 2.77 |
| Cys | Y=0.0001X-1.8507 | 0.995 | 2.5-62.5 | 0.41 | 2.62 |
| Tyr | Y=0.0002X-3.1099 | 0.997 | 5-125 | 0.41 | 3.57 |
| Val | Y=0.0002X-3.3077 | 0.996 | 5-125 | 0.43 | 2.42 |
| Met | Y=0.0002X-2.9627 | 0.997 | 5-125 | 0.70 | 1.30 |
| Lys | Y=0.0001X-3.3709 | 0.996 | 5-125 | 0.31 | 0.70 |
| Ile | Y=0.0002X-3.3097 | 0.996 | 5-125 | 0.48 | 2.66 |
| Leu | Y=0.0002X-4.0729 | 0.997 | 5-125 | 0.32 | 3.07 |
| Phe | Y=0.0002X-3.7141 | 0.998 | 5-125 | 0.49 | 4.25 |

Table S7 The content of 17 kinds of amino acids in 60 batches of *D. officinale* leaves (mg·g^-1^, n = 2)

| Varity | Month | Asp^△^ | Ser | Glu^△^ | Gly | His | Arg | Thr^*^ | Ala | Pro | Cys | Tyr | Val^*^ | Met^*^ | Lys^*^ | Ile^*^ | Leu^*^ | Phe^*^ |
| --- | --- | --- | --- | --- | --- | --- | --- | --- | --- | --- | --- | --- | --- | --- | --- | --- | --- | --- |
| Y2 | 10 | 8.52 | 5.32 | 10.68 | 6.37 | 2.54 | 6.59 | 5.38 | 5.98 | 5.68 | - | 5.29 | 6.38 | 0.26 | 4.93 | 5.97 | 10.41 | 7.41 |
| Y2 | 11 | 9.17 | 5.09 | 11.71 | 6.09 | 2.57 | 6.60 | 5.37 | 6.11 | 5.51 | - | 5.43 | 6.37 | 0.24 | 5.03 | 5.89 | 10.19 | 7.49 |
| Y2 | 12 | 7.86 | 4.85 | 10.76 | 6.53 | 1.07 | 6.67 | 5.36 | 7.65 | 5.47 | 0.10 | 2.78 | 7.20 | 0.38 | 4.11 | 6.83 | 11.16 | 4.23 |
| Y2 | 1 | 6.77 | 3.88 | 7.85 | 5.44 | 0.74 | 5.02 | 4.13 | 5.86 | 4.46 | - | 2.23 | 5.70 | 0.31 | 2.55 | 5.42 | 9.20 | 3.44 |
| Y2 | 2 | 6.35 | 3.40 | 7.66 | 5.29 | 0.77 | 4.63 | 3.80 | 5.60 | 4.15 | - | 1.98 | 5.30 | - | 2.24 | 5.06 | 8.63 | 3.04 |
| Y2 | 3 | 7.43 | 4.38 | 9.19 | 6.01 | 1.00 | 5.72 | 4.71 | 5.97 | 5.34 | - | 2.36 | 6.25 | - | 2.95 | 5.98 | 10.05 | 3.67 |
| Y2 | 4 | 8.66 | 5.52 | 11.16 | 7.20 | 1.20 | 6.73 | 5.66 | 6.94 | 5.81 | 0.05 | 2.74 | 7.29 | - | 4.45 | 6.80 | 11.52 | 4.25 |
| Y2 | 5 | 9.61 | 5.32 | 11.46 | 7.85 | 2.47 | 6.59 | 5.55 | 6.97 | 5.94 | 0.06 | 6.19 | 7.34 | 0.32 | 4.41 | 6.99 | 11.47 | 8.73 |
| Y2 | 6 | 7.36 | 4.25 | 8.83 | 5.66 | 1.86 | 4.69 | 4.11 | 5.29 | 4.41 | - | 4.40 | 5.24 | 0.07 | 3.50 | 4.93 | 8.47 | 6.55 |
| Y2 | 7 | 7.19 | 3.71 | 8.66 | 4.69 | 0.86 | 2.13 | 4.17 | 4.58 | 6.15 | - | 2.34 | 5.12 | 0.10 | 3.03 | 4.55 | 7.64 | 3.27 |
| Y2 | 8 | 5.85 | 3.30 | 6.69 | 4.44 | 0.82 | 2.08 | 4.17 | 3.91 | 5.44 | - | 2.29 | 4.58 | - | 2.56 | 4.11 | 6.72 | 3.02 |
| Y2 | 9 | 11.43 | 3.40 | 12.54 | 4.57 | 1.57 | 4.61 | 4.92 | 4.66 | 7.04 | - | 2.17 | 5.45 | 0.06 | 7.37 | 4.95 | 7.99 | 6.32 |
| B1 | 10 | 10.34 | 5.67 | 11.51 | 6.90 | 3.03 | 7.29 | 6.01 | 7.22 | 10.74 | 0.01 | 6.52 | 7.69 | 0.30 | 5.12 | 7.31 | 12.15 | 9.07 |
| B1 | 11 | 10.55 | 6.20 | 12.55 | 7.75 | 3.24 | 8.42 | 6.46 | 8.07 | 9.17 | 0.01 | 7.44 | 8.23 | 0.29 | 4.32 | 7.97 | 13.19 | 10.07 |
| B1 | 12 | 8.60 | 5.15 | 10.23 | 6.90 | 1.07 | 6.82 | 5.64 | 7.80 | 6.09 | 0.06 | 3.05 | 7.49 | 0.26 | 4.65 | 7.14 | 12.10 | 4.60 |
| B1 | 1 | 7.87 | 4.28 | 8.99 | 6.23 | 0.88 | 5.72 | 4.73 | 6.80 | 5.28 | 0.05 | 2.58 | 6.67 | 0.35 | 3.15 | 6.39 | 10.77 | 4.00 |
| B1 | 2 | 7.79 | 4.31 | 9.23 | 6.72 | 1.06 | 5.98 | 4.77 | 6.80 | 6.22 | 0.07 | 2.57 | 6.61 | 0.02 | 2.84 | 6.33 | 10.89 | 3.95 |
| B1 | 3 | 8.15 | 4.96 | 9.94 | 6.71 | 1.14 | 6.38 | 5.18 | 6.50 | 5.56 | 0.04 | 2.64 | 6.88 | - | 3.19 | 6.61 | 11.27 | 4.14 |
| B1 | 4 | 7.66 | 4.92 | 9.58 | 6.43 | 1.05 | 6.03 | 5.09 | 6.02 | 5.39 | - | 2.68 | 6.53 | - | 3.78 | 6.14 | 10.62 | 4.13 |
| B1 | 5 | 9.49 | 5.74 | 11.30 | 7.57 | 2.79 | 7.05 | 6.13 | 7.72 | 6.67 | 0.10 | 7.11 | 8.14 | 0.31 | 4.81 | 7.70 | 12.88 | 10.17 |
| B1 | 6 | 9.55 | 5.40 | 10.80 | 6.98 | 2.59 | 6.25 | 5.39 | 7.13 | 5.79 | 0.03 | 6.00 | 6.83 | 0.20 | 4.69 | 6.34 | 11.36 | 9.00 |
| B1 | 7 | 8.43 | 4.07 | 11.21 | 4.67 | 1.16 | 2.11 | 4.70 | 4.61 | 6.75 | - | 2.69 | 5.53 | - | 2.67 | 4.78 | 7.61 | 4.01 |
| B1 | 8 | 5.00 | 2.96 | 5.30 | 4.08 | 0.89 | 1.82 | 3.46 | 3.36 | 5.06 | - | 2.37 | 3.93 | - | 1.93 | 3.46 | 6.06 | 3.07 |
| B1 | 9 | 10.78 | 3.96 | 11.04 | 5.30 | 1.55 | 5.24 | 5.41 | 4.48 | 7.30 | - | 2.78 | 5.46 | 0.05 | 6.11 | 4.92 | 8.42 | 7.99 |
| B3 | 10 | 9.62 | 5.53 | 11.22 | 6.63 | 2.66 | 6.64 | 5.84 | 7.00 | 8.86 | - | 5.82 | 7.20 | 0.12 | 4.76 | 6.93 | 11.31 | 8.23 |
| B3 | 11 | 9.65 | 5.49 | 11.35 | 6.55 | 2.91 | 6.59 | 6.14 | 7.37 | 7.68 | - | 6.31 | 7.26 | 0.22 | 4.25 | 6.92 | 11.33 | 8.49 |
| B3 | 12 | 9.35 | 5.51 | 11.04 | 7.20 | 1.17 | 7.17 | 6.01 | 8.67 | 6.69 | 0.16 | 3.17 | 8.05 | 0.40 | 5.18 | 7.59 | 12.95 | 4.68 |
| B3 | 1 | 8.64 | 4.76 | 9.60 | 6.32 | 0.97 | 6.12 | 5.01 | 6.94 | 5.53 | 0.08 | 2.75 | 6.90 | 0.37 | 2.93 | 6.66 | 11.20 | 4.15 |
| B3 | 2 | 6.74 | 3.71 | 8.18 | 5.63 | 0.81 | 4.79 | 4.00 | 6.27 | 5.10 | - | 2.10 | 5.67 | - | 2.39 | 5.44 | 9.30 | 3.30 |
| B3 | 3 | 9.18 | 5.54 | 11.53 | 7.52 | 1.25 | 7.05 | 5.68 | 7.54 | 6.21 | 0.03 | 2.90 | 7.79 | - | 4.09 | 7.41 | 12.64 | 4.43 |
| B3 | 4 | 7.89 | 5.13 | 9.93 | 6.48 | 1.06 | 6.14 | 5.12 | 6.26 | 5.40 | 0.05 | 2.57 | 6.62 | - | 3.67 | 6.28 | 10.73 | 3.95 |
| B3 | 5 | 10.08 | 5.97 | 12.21 | 8.24 | 2.76 | 7.05 | 6.02 | 7.83 | 6.55 | 0.08 | 6.48 | 7.92 | 0.33 | 5.01 | 7.50 | 12.49 | 9.38 |
| B3 | 6 | 9.03 | 5.16 | 10.41 | 6.30 | 2.38 | 5.94 | 5.04 | 6.59 | 5.40 | 0.05 | 5.47 | 6.57 | 0.23 | 4.47 | 6.17 | 10.57 | 7.97 |
| B3 | 7 | 9.33 | 3.70 | 8.98 | 4.93 | 0.95 | 2.28 | 4.50 | 5.27 | 6.28 | - | 2.44 | 5.70 | - | 3.48 | 4.99 | 8.18 | 3.53 |
| B3 | 8 | 8.15 | 4.00 | 8.56 | 5.12 | 0.97 | 2.48 | 5.11 | 5.13 | 6.72 | - | 2.49 | 5.59 | 0.04 | 3.71 | 4.92 | 8.17 | 3.28 |
| B3 | 9 | 13.06 | 4.71 | 15.53 | 6.13 | 1.54 | 6.07 | 5.90 | 6.15 | 8.34 | - | 0.03 | 6.91 | 0.50 | 7.81 | 6.21 | 10.10 | 9.18 |
| 630 | 10 | 13.44 | 7.48 | 15.46 | 8.89 | 4.00 | 9.45 | 8.10 | 9.95 | 11.55 | 0.08 | 8.21 | 10.13 | 0.55 | 6.73 | 9.47 | 15.65 | 11.51 |
| 630 | 11 | 11.10 | 6.19 | 13.34 | 7.72 | 3.29 | 8.65 | 6.62 | 8.48 | 9.08 | 0.02 | 7.06 | 8.55 | 0.47 | 4.78 | 8.08 | 13.35 | 9.98 |
| 630 | 12 | 10.64 | 6.28 | 12.79 | 8.53 | 1.48 | 8.61 | 6.99 | 9.87 | 7.40 | 0.13 | 3.70 | 9.26 | 0.54 | 5.68 | 8.75 | 14.74 | 5.57 |
| 630 | 1 | 8.99 | 5.17 | 10.73 | 7.21 | 1.14 | 6.77 | 5.61 | 8.03 | 5.93 | 0.05 | 3.01 | 7.75 | 0.54 | 3.56 | 7.37 | 12.51 | 4.57 |
| 630 | 2 | 8.10 | 4.55 | 9.76 | 6.82 | 1.14 | 5.92 | 4.95 | 6.87 | 5.83 | 0.04 | 2.61 | 6.77 | 0.08 | 2.91 | 6.46 | 11.17 | 4.06 |
| 630 | 3 | 9.50 | 5.55 | 11.68 | 7.71 | 1.35 | 7.37 | 5.99 | 8.06 | 6.38 | 0.02 | 3.10 | 8.15 | 0.05 | 4.04 | 7.69 | 13.02 | 4.81 |
| 630 | 4 | 8.11 | 4.94 | 10.04 | 6.50 | 1.06 | 6.28 | 5.21 | 6.70 | 5.39 | 0.03 | 2.68 | 6.77 | - | 3.58 | 6.36 | 10.79 | 4.09 |
| 630 | 5 | 12.74 | 7.27 | 15.34 | 10.26 | 3.68 | 8.93 | 7.68 | 10.00 | 8.23 | 0.15 | 8.34 | 10.24 | 0.58 | 6.07 | 9.62 | 16.10 | 12.14 |
| 630 | 6 | 11.62 | 7.19 | 13.82 | 9.24 | 3.72 | 8.77 | 7.23 | 9.25 | 8.58 | 0.10 | 7.61 | 9.22 | 0.42 | 6.27 | 8.41 | 14.64 | 11.54 |
| 630 | 7 | 9.30 | 5.31 | 12.91 | 5.52 | 1.37 | 2.79 | 5.73 | 5.66 | 7.51 | - | 2.98 | 6.99 | 0.08 | 3.95 | 6.07 | 9.58 | 4.47 |
| 630 | 8 | 8.12 | 4.17 | 8.10 | 5.39 | 1.10 | 2.66 | 5.03 | 5.19 | 6.45 | - | 2.73 | 5.65 | 0.02 | 3.30 | 4.97 | 8.14 | 3.67 |
| 630 | 9 | 15.53 | 4.66 | 13.97 | 6.06 | 1.61 | 6.52 | 6.48 | 5.93 | 8.16 | - | 3.39 | 6.72 | 0.38 | 7.38 | 6.02 | 9.82 | 9.72 |
| Y3 | 10 | 7.92 | 4.78 | 9.95 | 5.78 | 2.34 | 6.07 | 4.89 | 6.41 | 7.47 | - | 5.30 | 6.39 | 0.12 | 4.12 | 5.99 | 9.75 | 7.33 |
| Y3 | 11 | 8.01 | 4.72 | 9.48 | 5.90 | 2.38 | 5.70 | 5.39 | 6.04 | 6.70 | - | 5.28 | 6.15 | 0.12 | 3.56 | 5.91 | 9.92 | 7.63 |
| Y3 | 12 | 8.04 | 4.84 | 9.63 | 6.59 | 1.01 | 6.35 | 5.27 | 6.94 | 5.70 | 0.04 | 2.79 | 6.95 | 0.24 | 4.34 | 6.67 | 11.37 | 4.33 |
| Y3 | 1 | 7.38 | 4.38 | 8.79 | 6.15 | 0.80 | 5.59 | 4.60 | 6.05 | 5.01 | 0.03 | 2.45 | 6.33 | 0.22 | 2.50 | 6.23 | 10.37 | 3.86 |
| Y3 | 2 | 7.14 | 4.15 | 8.63 | 6.04 | 0.93 | 5.30 | 4.36 | 6.16 | 4.88 | 0.04 | 2.29 | 5.93 | - | 2.35 | 5.69 | 9.91 | 3.63 |
| Y3 | 3 | 7.68 | 4.91 | 9.39 | 6.70 | 1.08 | 6.11 | 5.05 | 6.12 | 5.46 | - | 2.67 | 6.76 | - | 3.29 | 6.46 | 11.00 | 4.21 |
| Y3 | 4 | 10.95 | 6.63 | 13.52 | 8.35 | 1.55 | 8.57 | 6.98 | 8.43 | 7.18 | 0.06 | 3.75 | 8.90 | 0.02 | 5.43 | 8.50 | 14.85 | 5.71 |
| Y3 | 5 | 9.76 | 5.62 | 11.63 | 7.83 | 2.71 | 6.64 | 5.92 | 7.32 | 6.58 | 0.06 | 6.56 | 7.88 | 0.23 | 4.93 | 7.48 | 12.54 | 9.65 |
| Y3 | 6 | 8.23 | 4.93 | 9.78 | 6.41 | 2.36 | 5.81 | 4.98 | 6.06 | 5.26 | 0.02 | 5.42 | 6.36 | 0.17 | 4.42 | 5.93 | 10.38 | 8.09 |
| Y3 | 7 | 8.50 | 4.30 | 9.35 | 5.70 | 1.12 | 2.68 | 5.07 | 5.81 | 6.56 | - | 2.91 | 6.17 | - | 3.45 | 5.55 | 9.11 | 3.99 |
| Y3 | 8 | 6.73 | 3.95 | 7.54 | 5.34 | 1.00 | 2.44 | 4.98 | 4.56 | 6.69 | - | 2.69 | 5.35 | - | 3.14 | 4.82 | 8.20 | 3.61 |
| Y3 | 9 | 8.74 | 3.08 | 9.94 | 4.28 | 1.01 | 4.21 | 4.61 | 3.86 | 6.38 | - | 2.11 | 4.75 | - | 5.82 | 4.18 | 6.97 | 6.27 |

-: not detected; *: essential amino acids; △: umami taste amino acid

Table S8 Vitamin content of 60 batches of *D. officinale* leaves (μg·g^-^^1^, n = 2)

| Varity | Month | Vitamin B_5_ | Vitamin B_1_ | NMN | Vitamin B_2_ | Folic acid |
| --- | --- | --- | --- | --- | --- | --- |
| Y2 | 10 | 17.01 | 6.86 | 3.31 | 2.68 | 9.11 |
| Y2 | 11 | 1.68 | 2.90 | 12.51 | 7.71 | 0.25 |
| Y2 | 12 | 1.14 | 1.47 | 6.49 | 5.85 | 2.78 |
| Y2 | 1 | 0.80 | 1.36 | 1.59 | 6.09 | 2.67 |
| Y2 | 2 | 0.53 | 2.46 | - | 4.31 | 0.43 |
| Y2 | 3 | - | 2.58 | - | 3.93 | 0.18 |
| Y2 | 4 | 0.91 | 3.33 | 0.27 | 4.65 | 0.43 |
| Y2 | 5 | 0.92 | 2.47 | 0.09 | 2.96 | - |
| Y2 | 6 | 0.70 | 2.84 | 0.96 | 3.07 | 0.51 |
| Y2 | 7 | 1.37 | 1.89 | 0.34 | 3.55 | 0.68 |
| Y2 | 8 | 0.36 | 2.37 | 0.80 | 7.81 | 0.36 |
| Y2 | 9 | 0.14 | 1.94 | 0.02 | 5.94 | 2.35 |
| B1 | 10 | 31.65 | 8.59 | - | 3.97 | 1.27 |
| B1 | 11 | 16.15 | 9.27 | - | 5.09 | 4.23 |
| B1 | 12 | 32.30 | 10.41 | 1.96 | 3.96 | 2.34 |
| B1 | 1 | 1.00 | 1.02 | - | 3.65 | 1.01 |
| B1 | 2 | 0.71 | 2.03 | - | 3.92 | 0.29 |
| B1 | 3 | 0.83 | 1.90 | 0.22 | 2.25 | 2.04 |
| B1 | 4 | 0.59 | 2.62 | 0.08 | 4.97 | 0.02 |
| B1 | 5 | 0.82 | 1.74 | 0.01 | 2.77 | - |
| B1 | 6 | 1.72 | 3.20 | 0.31 | 4.32 | 0.11 |
| B1 | 7 | 3.32 | 1.26 | 0.33 | 3.90 | 0.27 |
| B1 | 8 | 0.46 | 1.83 | 0.80 | 5.74 | 0.20 |
| B1 | 9 | 0.36 | 2.06 | - | 5.82 | 2.18 |
| B3 | 10 | 21.49 | 8.65 | - | 3.16 | 2.62 |
| B3 | 11 | 19.84 | 8.04 | 1.95 | 4.77 | 1.53 |
| B3 | 12 | 0.79 | 2.09 | 0.68 | 5.54 | 0.24 |
| B3 | 1 | 0.36 | 1.27 | - | 4.53 | 0.99 |
| B3 | 2 | 0.52 | 1.99 | 0.50 | 7.38 | 0.05 |
| B3 | 3 | - | 2.00 | - | 3.36 | 0.18 |
| B3 | 4 | 0.80 | 2.40 | 0.11 | 3.46 | - |
| B3 | 5 | 2.12 | 2.41 | 0.32 | 2.72 | - |
| B3 | 6 | 1.57 | 2.43 | 0.31 | 3.06 | 0.05 |
| B3 | 7 | 2.12 | 1.15 | 0.34 | 3.55 | 0.21 |
| B3 | 8 | 0.97 | 1.78 | 0.81 | 6.46 | 0.17 |
| B3 | 9 | 0.91 | 1.98 | - | 6.93 | 2.15 |
| 630 | 10 | 32.15 | 11.85 | - | 4.33 | 0.08 |
| 630 | 11 | 0.43 | 2.95 | 0.36 | 6.35 | 0.05 |
| 630 | 12 | 21.33 | 10.42 | 2.01 | 5.77 | 2.49 |
| 630 | 1 | 0.61 | 0.87 | - | 3.70 | 0.98 |
| 630 | 2 | 0.68 | 1.93 | 1.62 | 14.95 | 2.65 |
| 630 | 3 | - | 2.36 | - | 3.87 | 0.16 |
| 630 | 4 | 0.36 | 2.79 | 0.22 | 5.05 | - |
| 630 | 5 | 0.66 | 2.31 | 0.40 | 3.40 | - |
| 630 | 6 | 0.54 | 4.09 | 0.39 | 5.23 | 0.03 |
| 630 | 7 | 3.22 | 1.57 | 0.61 | 4.63 | 0.21 |
| 630 | 8 | 0.74 | 2.00 | 0.88 | 7.46 | 0.14 |
| 630 | 9 | 0.58 | 2.83 | 0.14 | 7.10 | 2.14 |
| Y3 | 10 | 34.87 | 7.06 | - | 3.54 | 0.74 |
| Y3 | 11 | 12.08 | 12.76 | 1.95 | 5.70 | 3.39 |
| Y3 | 12 | 31.61 | 8.62 | 1.99 | 3.63 | 1.84 |
| Y3 | 1 | 0.55 | 0.88 | - | 2.92 | 0.98 |
| Y3 | 2 | 0.85 | 1.97 | - | 3.32 | 0.23 |
| Y3 | 3 | - | 2.12 | - | 3.01 | 0.17 |
| Y3 | 4 | 0.27 | 2.54 | 0.12 | 2.62 | - |
| Y3 | 5 | 0.61 | 2.02 | 0.09 | 2.47 | - |
| Y3 | 6 | 0.66 | 3.37 | 0.38 | 3.28 | 0.01 |
| Y3 | 7 | 1.51 | 1.55 | 0.30 | 4.35 | 0.20 |
| Y3 | 8 | 0.30 | 2.30 | 0.80 | 5.78 | 0.14 |
| Y3 | 9 | 0.24 | 2.38 | 0.06 | 4.47 | 2.12 |

-: not detected


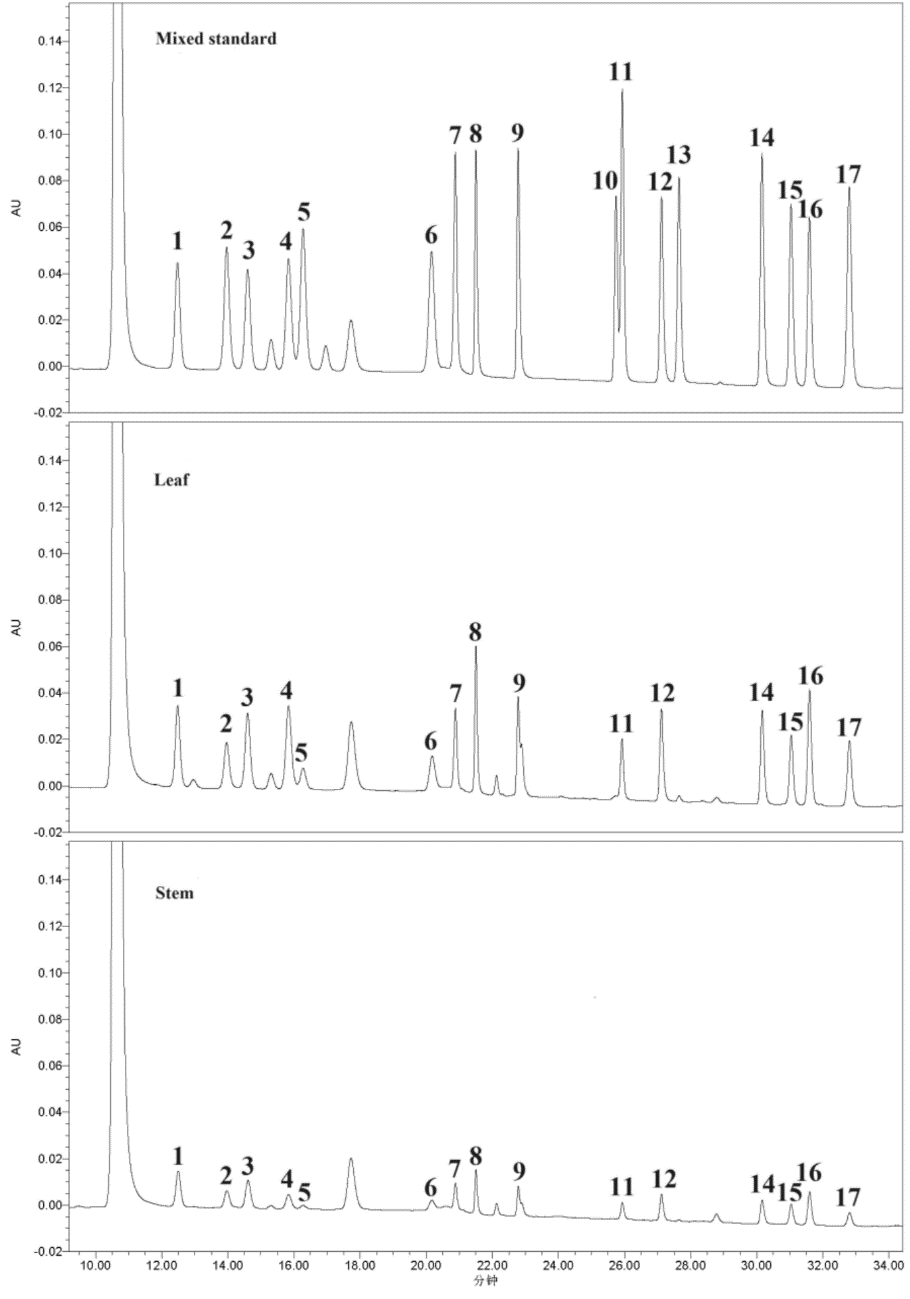


Fig. S1 Amino acid determination chromatogram (1: Asp; 2:Ser; 3: Glu; 4: Gly; 5: His; 6: Arg; 7: Thr; 8: Ala; 9: Pro; 10: Cys; 11: Tyr; 12: Val; 13: Met; 14: Lys; 15: Ile; 16: Leu; 17: Phe)


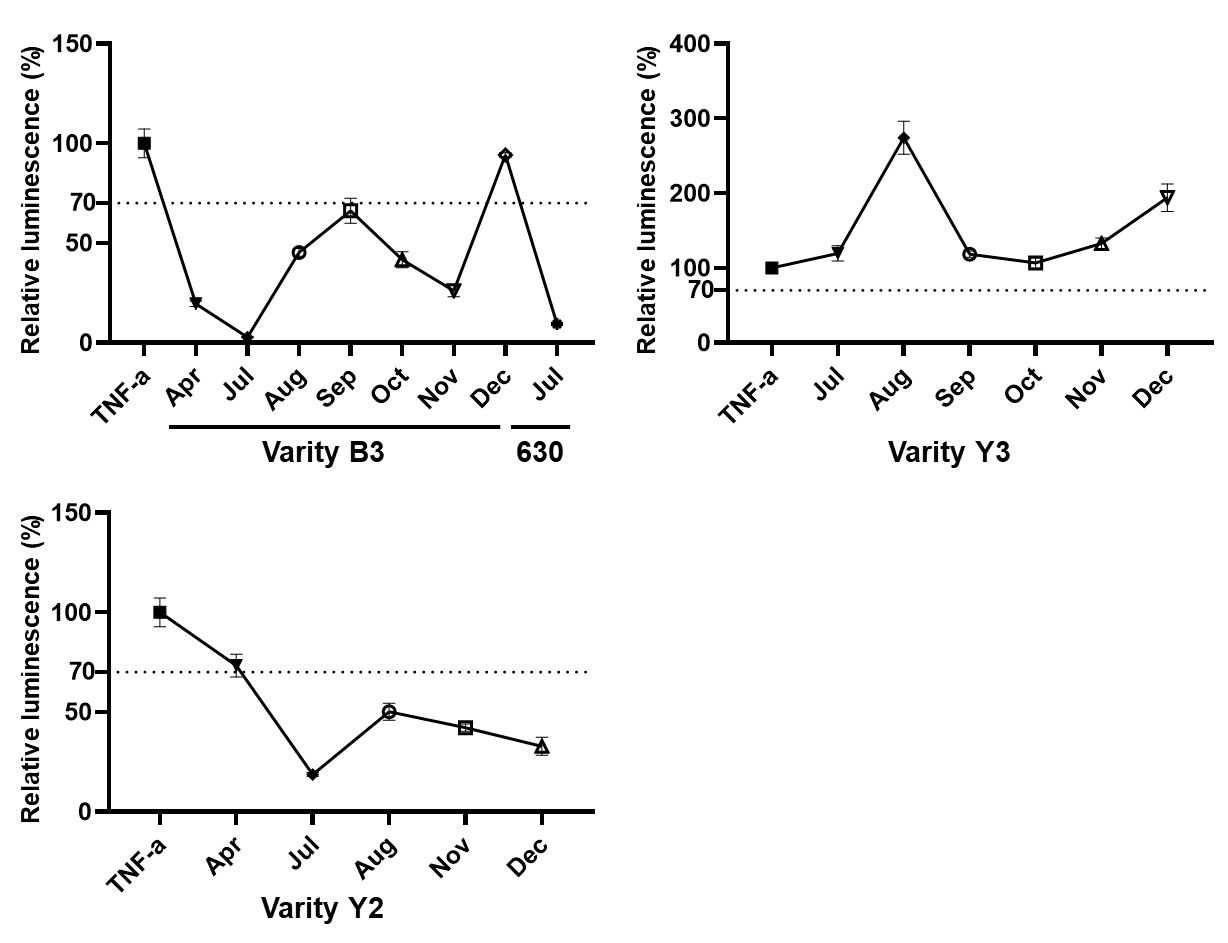


Fig. S2. In vitro validation results of anti-inflammatory activity


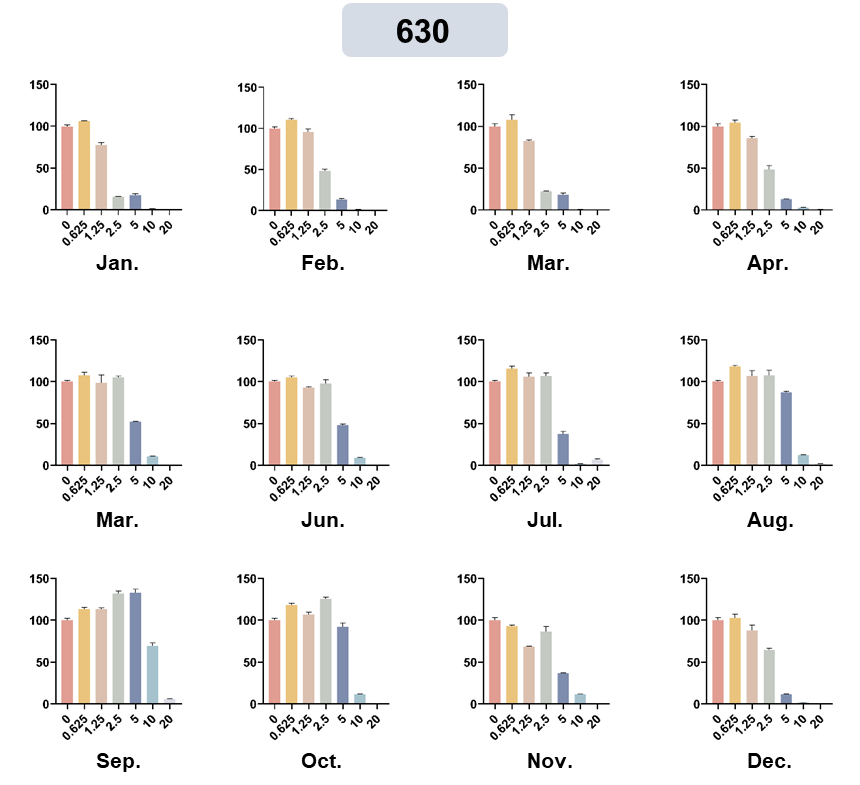


Fig. S3 Effect of variety 630 on the activity of HEK-293T-NFkB cells


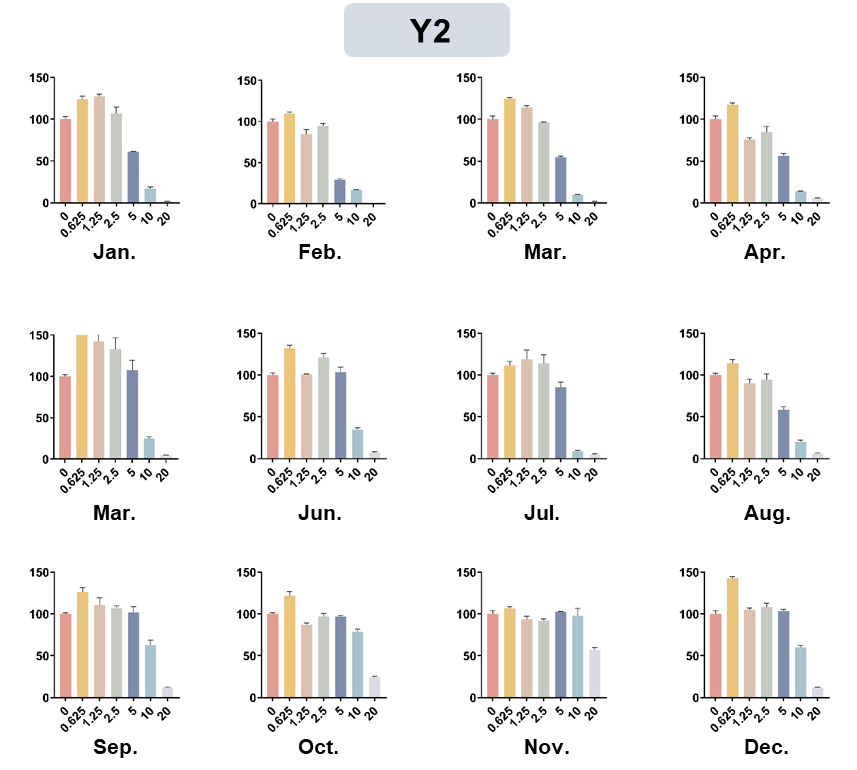


Fig. S4 Effect of variety Y2 on the activity of HEK-293T-NFkB cells


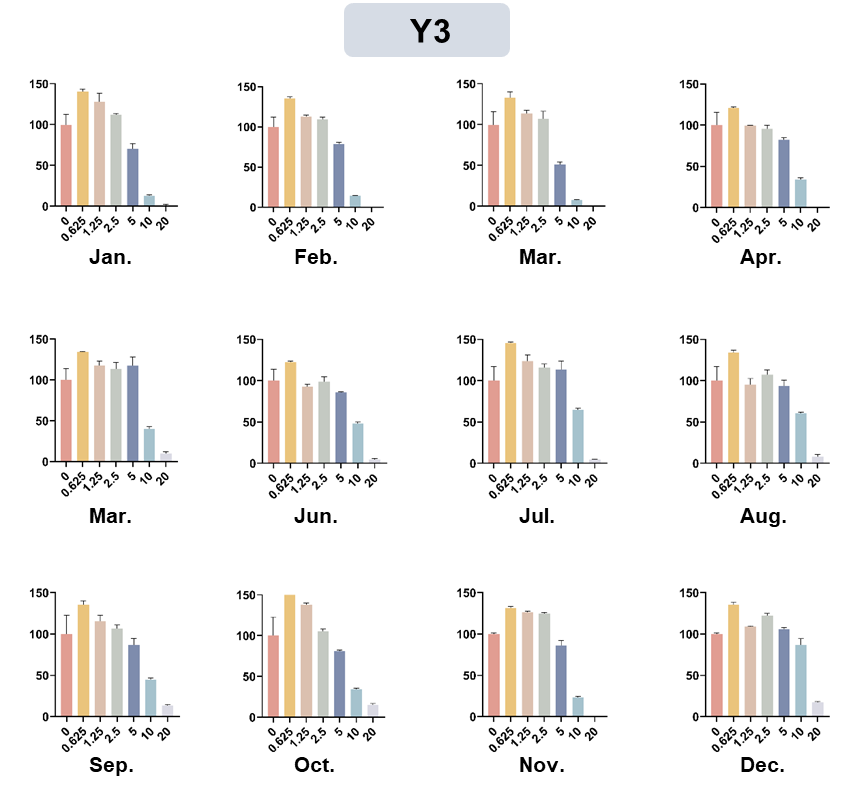


Fig. S5 Effect of variety Y3 on the activity of HEK-293T-NFkB cells


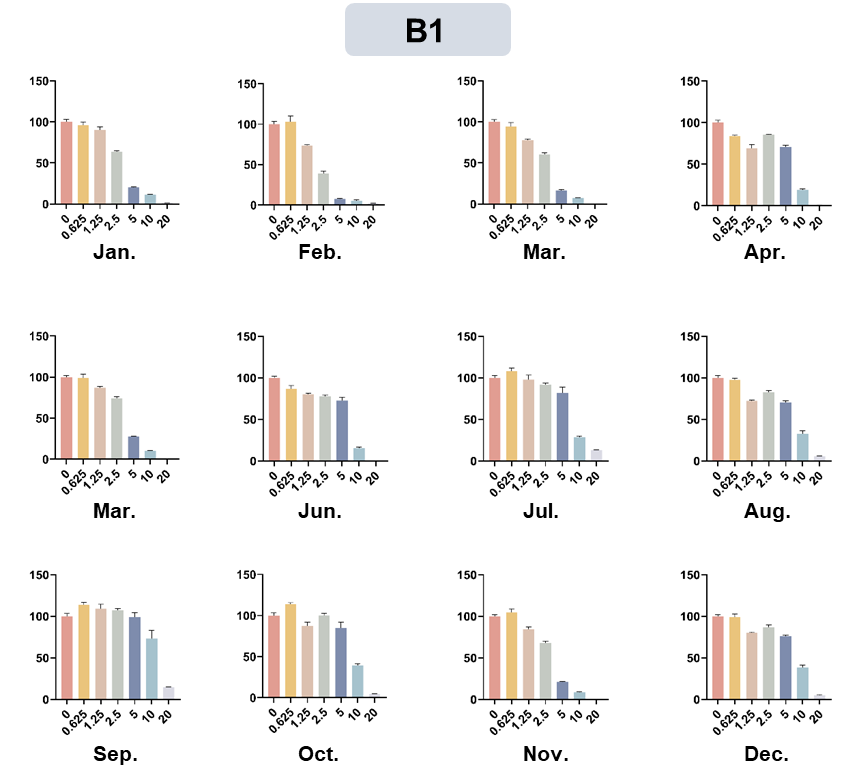


Fig. S6 Effect of variety B1 on the activity of HEK-293T-NFkB cells


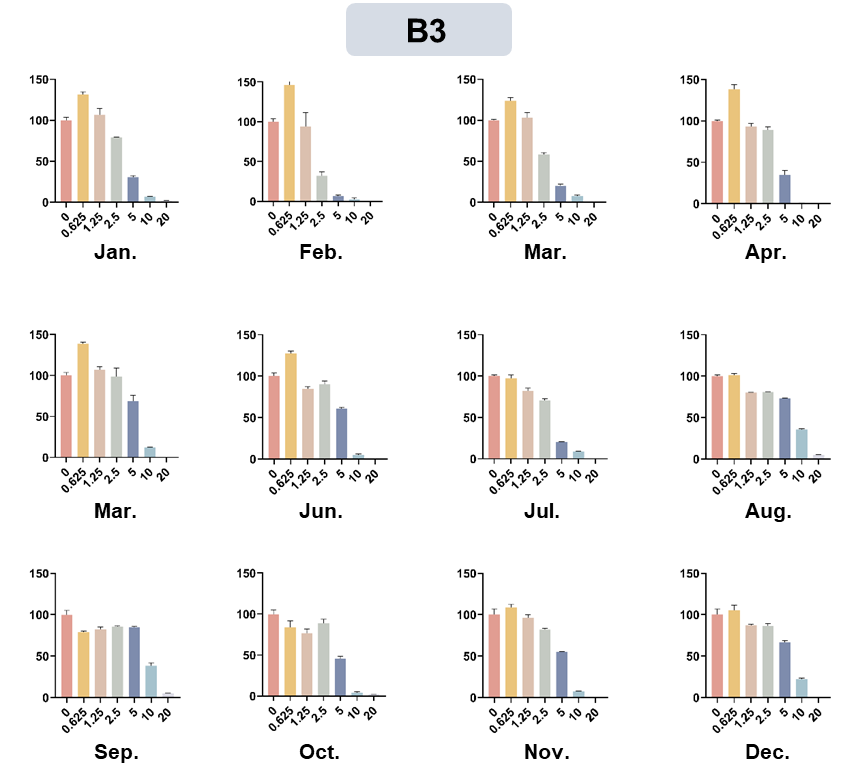


Fig. S7 Effect of variety B3 on the activity of HEK-293T-NFkB cells
